# Validation of an Echidna Forelimb Musculoskeletal Model Using XROMM and diceCT

**DOI:** 10.3389/fbioe.2021.751518

**Published:** 2021-11-08

**Authors:** Sophie Regnault, Philip Fahn-Lai, Stephanie E. Pierce

**Affiliations:** ^1^ Museum of Comparative Zoology and Department of Organismic and Evolutionary Biology, Harvard University, Cambridge, MA, United States; ^2^ Institute of Biological, Environment and Rural Sciences, Aberystwyth University, Aberystwyth, United Kingdom; ^3^ Concord Field Station and Department of Organismic and Evolutionary Biology, Harvard University, Bedford, MA, United States

**Keywords:** muscle moment arm, range of motion, SIMM, joint, mobility, translation, biomechanics, muscle

## Abstract

In evolutionary biomechanics, musculoskeletal computer models of extant and extinct taxa are often used to estimate joint range of motion (ROM) and muscle moment arms (MMAs), two parameters which form the basis of functional inferences. However, relatively few experimental studies have been performed to validate model outputs. Previously, we built a model of the short-beaked echidna (*Tachyglossus aculeatus*) forelimb using a traditional modelling workflow, and in this study we evaluate its behaviour and outputs using experimental data. The echidna is an unusual animal representing an edge-case for model validation: it uses a unique form of sprawling locomotion, and possesses a suite of derived anatomical features, in addition to other features reminiscent of extinct early relatives of mammals. Here we use diffusible iodine-based contrast-enhanced computed tomography (diceCT) alongside digital and traditional dissection to evaluate muscle attachments, modelled muscle paths, and the effects of model alterations on the MMA outputs. We use X-ray Reconstruction of Moving Morphology (XROMM) to compare *ex vivo* joint ROM to model estimates based on osteological limits predicted via single-axis rotation, and to calculate experimental MMAs from implanted muscles using a novel geometric method. We also add additional levels of model detail, in the form of muscle architecture, to evaluate how muscle torque might alter the inferences made from MMAs alone, as is typical in evolutionary studies. Our study identifies several key findings that can be applied to future models. 1) A light-touch approach to model building can generate reasonably accurate muscle paths, and small alterations in attachment site seem to have minimal effects on model output. 2) Simultaneous movement through multiple degrees of freedom, including rotations and translation at joints, are necessary to ensure full joint ROM is captured; however, single-axis ROM can provide a reasonable approximation of mobility depending on the modelling objectives. 3) Our geometric method of calculating MMAs is consistent with model-predicted MMAs calculated via partial velocity, and is a potentially useful tool for others to create and validate musculoskeletal models. 4) Inclusion of muscle architecture data can change some functional inferences, but in many cases reinforced conclusions based on MMA alone.

## Introduction

Three-dimensional musculoskeletal computer models have become widely used to test hypotheses of biomechanical function in both extant and extinct animals. Such models are increasingly used to infer species-specific functional parameters (e.g., Pierce et al., 2012; Demuth et al., 2020; Bishop et al., 2021b; Richards et al., 2021), as well as in larger scale comparative analyses to characterise trends in functional evolution (e.g., Bates et al., 2012; Allen et al., 2021; Molnar et al., 2021). Two fundamental parameters of interest to evolutionary and biomechanical researchers are joint range of motion (ROM) and muscle moment arms (MMAs). A major advantage of 3D models is that they allow analyses of these parameters through non-planar motions, and so expand the types of movements and animals that can be studied (e.g., Regnault and Pierce, 2018). Models also provide a means to collect difficult-to-acquire data in extant animals (e.g., due to specimen scarcity or requiring invasive collection techniques) and, more recently, to more rigorously explore functional reconstruction in extinct animals (e.g., Hutchinson, 2012; Brassey et al., 2017; Nyakatura et al., 2019; Bishop et al., 2021c). However, there is a recognised gap – particularly in paleobiology – between the number of modelling studies published versus validation studies (Brassey et al., 2017), despite acceptance that such validation is critical to evaluate model-building practices and appropriately interpret results.

ROM estimates in extinct animals can eliminate improbable poses to constrain hypotheses about the types of mobility achievable (Gatesy et al., 2009; Manafzadeh and Padian, 2018; Manafzadeh et al., 2021) and so can inform understanding of major evolutionary transitions (e.g., water-to-land in tetrapods; Pierce et al., 2012). Where experimental data are not available, model ROM estimates are usually made through digital manipulation of bones until bone-on-bone contact or probable joint disarticulation (e.g., Mallison, 2010; Pierce et al., 2012; Lai et al., 2018; Manafzadeh and Padian, 2018; Bishop et al., 2021a; Richards et al., 2021). Traditionally, this has been done for each rotational degree of freedom (DOF) independently i.e., flexion-extension, abduction-adduction, long-axis rotation. More recently, Manafzadeh and Padian (2018) developed a semi-automated workflow that allows simultaneous rotations through each DOF to calculate an “envelope” of movement. As well as interactions between rotational DOF, other factors have also been long-identified as relevant to model-predictions of ROM: the effect of missing soft tissues, joint spacing, and translation at joint surfaces. Soft tissues can limit ROM directly (e.g., ligament, joint capsule) or indirectly (e.g., muscle bulk, integument) and several studies have documented the effects of different soft tissues (e.g., Hutson and Hutson, 2012; Pierce et al., 2012; Arnold et al., 2014), but an explicit methodology for applying these observations to extinct animal reconstructions is lacking (Manafzadeh and Padian, 2018). Joint spacing can alter ROM estimates (e.g., Regnault and Pierce, 2018), and can be difficult to account for in disarticulated specimens; in fossils, estimates are often made from the intra-articular distances and cartilage morphologies of living relatives (e.g., Holliday et al., 2010; Molnar et al., 2021). Translations at joint surfaces can greatly increase ROM (Pierce et al., 2012; Manafzadeh and Gatesy, 2021) but can be difficult to implement simultaneously with rotational DOF, and are often excluded from models for simplicity. Previous studies that include joint translations have programmed it as a pre-defined function coupled to specific joint rotations (Pierce et al., 2012; Richards et al., 2021) or account for translation by iteratively adjusting the starting (reference) position of the distal bone prior to performing joint rotations (Arnold et al., 2014; Manafzadeh and Padian, 2018).

Moment arms give an indication of a muscle’s leverage, or effectiveness at generating specific rotational forces at joints (Sherman et al., 2013). MMAs are crucial for understanding how muscles produce (or resist) movement (Brassey et al., 2017), from the level of individual muscle role (e.g., flexor vs. extensor; Regnault and Pierce, 2018) to whole animal function (Fujiwara and Hutchinson, 2012; Bates et al., 2012; Bishop et al., 2021c; Wiseman et al., 2021), to comparative function between animals (e.g., evolutionary trends; Maidment et al., 2014; Molnar et al., 2021; Allen et al., 2021). MMAs are also the basis for calculating further parameters of interest; for example, combined with muscle architecture and kinematic data to calculate muscle and joint torques. MMAs are known to change with joint position and limb orientation (An et al., 1984), and so 3D models are an ideal way to study functional consequences at multiple scale-levels previously mentioned (from individual muscles to evolutionary trends), provided that models have been well-validated in the context in which results are interpreted. In human clinical biomechanics, MMAs have been validated against experimental data but such validation studies are relatively rare outside of humans and other bipedal and parasagittal animals (Kargo and Rome, 2002), and for muscles crossing complex joints (for example, a single biological “joint” comprising several bony articulations, or exhibiting coupled motions; Sherman et al., 2013; Brassey et al., 2017). A further consideration for validation is the several ways to measure MMA: estimates are commonly made from either tendon-travel or geometric measurements, around fixed (anatomical) or moving (kinematic) joint centres and axes. The equivalence of different methods is unclear, and possibly a source of variation when attempting to validate MMAs acquired through different means. The use of several methods has been advocated as a cross-check, where possible (An et al., 1984). The scope of validation may differ depending on the purpose of the study; for example, absolute values for specific behaviours vs. relative trends across taxa.

In a previous study (Regnault and Pierce, 2018), we built a musculoskeletal model of a short-beaked echidna (Monotremata: *Tachyglossus aculeatus*) forelimb to investigate osteological joint ROM and MMAs. We took a traditional model-building approach, commonly used to model extinct animals, to identify learning opportunities that could be applied to future models of extinct synapsids in studying the evolution of the mammalian forelimb. Here, we aim to critically evaluate the behaviour and outputs from the initial echidna musculoskeletal model against experimental data, taking into account some of the considerations for ROM and MMA predictions mentioned above. In the initial model, ROMs were predicted through 1 DOF independent rotations around an anatomical joint centre until bone-bone contact. Muscle geometry was modelled as lines of action between bony attachment sites, with the minimal ‘wrapping’ needed to prevent muscles from passing through bones. MMAs were calculated using a partial velocity method (equivalent to tendon travel) through each rotational DOF independently. In the current study, we now seek to validate and refine the model via several stages: 1) contrast stain (via diceCT) and digitally dissect the echidna specimen’s forelimb muscles to evaluate the accuracy of a minimalist wrapping approach in replicating muscle geometry, and evaluate the effect of inaccuracies on predicted MMA; 2) collect maximal ROM data via passive manipulation of cadavers using bi-planar x-ray fluoroscopes and X-ray Reconstruction of Moving Morphology (XROMM; Brainerd et al., 2010) to evaluate single-axis rotational DOF model predictions against experimental joint excursions in both rotation and translation (up to 6 DOF); and 3) develop a geometric method of calculating MMA from markers implanted in cadaver muscles, to evaluate both model-predicted MMAs and different methods of calculating MMAs. We also combine MMA from the initial model with muscle architecture data (Regnault et al., 2020) to evaluate the functional interpretations that can be made from MMAs alone (typical outputs of extinct animal models) versus more holistic parameters (muscle torque).

## Materials and Methods

**FIGURE 1:**
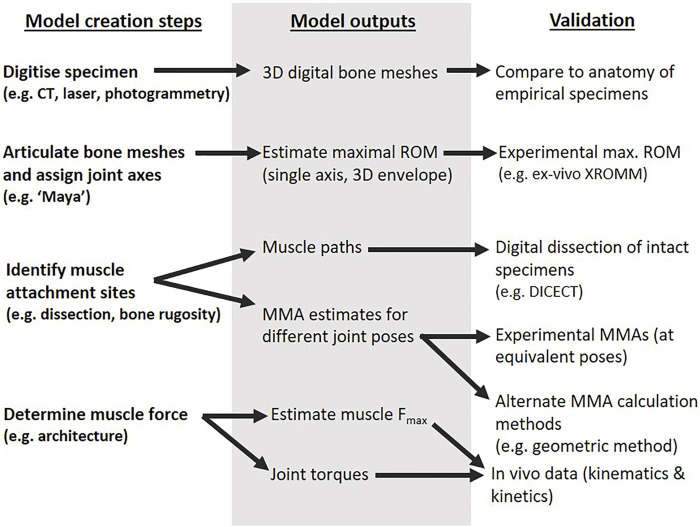
Different components of the musculoskeletal modelling process as detailed in the text.

The typical components of the musculoskeletal modelling process are outlined in Figure 1 (Bishop, 2021a), from model creation to outputs and validation using experimental data. The steps are often iterative, and validation of a previous step may feed into subsequent model creation and output (though care must be taken to avoid circularity or targeted results by specifying the methods, acceptable adjustments, and rationale for each *a priori*). The detailed methods for the creation steps of our echidna forelimb model (left column of Figure 1) are described in Regnault and Pierce (2018) and Regnault et al. (2020). In this study, we critically examine our model outputs through diceCT, *ex vivo* XROMM, and details of muscle architecture.

### Digital Dissection and Evaluation of Muscle Paths

The initial musculoskeletal model (Regnault and Pierce, 2018) was built using the modelling software SIMM (Delp and Loan, 1995). The model was created following the steps of Figure 1: bone meshes were obtained from computed tomography (CT) scans of an echidna cadaver (first row of Figure 1), articulated with anatomical joint axes based on shape “primitives” fitted to joint surfaces (second row of Figure 1), and used muscle attachment sites identified from the qualitative descriptions of Gambaryan et al. (2015) (third row of Figure 1). To enable specimen-specific evaluation of muscle attachments and muscle paths in the model and ensure model accuracy (right column of Figure 1), the same echidna specimen underwent digital dissection (Gignac et al., 2016). The specimen was contrast-stained with a 3% iodine potassium iodide solution, and micro-CT scanned a second time to visualise its soft-tissue anatomy. The muscles were digitally segmented, and three-dimensional muscle meshes created to identify muscle attachment regions on the bones. The full method and illustrations of attachment regions are detailed in Regnault et al. (2020). No overt pathological changes or other anomalies were evident in the shoulder and forelimb region of this specimen or the others used in this study; all specimens were evaluated to confirm skeletal maturity (fused epiphyses) and lack of injury/pathology via radiography, computed tomography, dissection, and (for the modelled specimen) diceCT/digital dissection.

Muscle origin and insertion coordinates from the initial model were compared with the diceCT-identified attachment regions. Where the model’s muscle attachment coordinates did not fall within the diceCT-identified regions, model coordinates were adjusted within SIMM. The effects of adjusting muscle attachment coordinates on model-estimated MMAs are detailed in the Results.

The initial model’s muscle pathways between origin and insertion were also compared to the diceCT muscle geometry. The digital bone and muscle meshes segmented from the diceCT scan were imported into SIMM, alongside the initial model. The initial model’s forelimb position was aligned, via rotation around its joint centres, to the forelimb position of the specimen in the diceCT scan. In this way, the model’s muscle lines of action could be compared to the actual muscle geometry for this position. Modelled muscles whose paths deviated appreciably from the digitally segmented muscle meshes were adjusted and the wrap objects associated with these muscles were edited. The effects of these adjustments on model-predicted MMAs are likewise reported in Results. The updated musculoskeletal model (with adjusted muscle attachments and pathways, based on diceCT) was used for all follow-on MMA and torque analyses.

### Experimental Set-Up and Data Collection

Experimental data were collected to validate model estimates of maximal joint ROMs and MMAs at the scapulocoracoid-clavicle-interclavicle, glenohumeral, and humeroradioulnar joints (second and third rows of Figure 1). Data collection consisted of passive manipulation of three echidna cadavers, using marker-based X-ray Reconstruction of Moving Morphology (XROMM; Brainerd et al., 2010). The echidna cadavers were obtained from the University of Adelaide (as per descriptions in Regnault et al., 2020), stored frozen at −18°C, and thawed at 4°C prior to data collection.

To track bone positions and calculate joint kinematics, 1 mm tantalum markers were implanted into holes predrilled into bones, using a 0.96 mm tungsten carbide hand drill. Three markers were implanted into each of the following bones (both left and right forelimb bones): the fused clavicle-interclavicle, scapulocoracoid, humerus, radius, and ulna. Subsequent animation and analysis showed the radius markers to display error associated with co-linearity, due to the size, shape and accessibility of the radius constraining marker placement sites. Because of this, we chose to animate the antebrachium as one unit (i.e., radius taking on the rotations and translations of the ulna). The radius and ulna generally move as a unit in the echidna, though some lateral displacement of the radius is possible (Haines, 1946). Our analyses therefore could not verify radial movement relative to the ulna, but were sufficient to evaluate elbow ROM. Two markers were also implanted into the sternum and one marker in the vertebral column, so that the body position could be approximated as a “body plane”.

To calculate experimental MMAs (third row of Figure 1), markers were also inserted into select muscles of each specimen based on their accessibility. A 16G needle was used to implant 0.8 mm tantalum markers into the following muscles: m. latissimus dorsi, m. pectoralis, m. triceps brachii (pars superficialis longus), m. biceps brachii, m. coracobrachialis (pars longus), and m. clavodeltoideus. Several other muscles were also implanted (m. triceps brachii pars lateralis and profundus, m. subscapularis), but later eliminated from analysis due to marker migration. A maximum of two muscles were implanted per specimen side (right or left) to facilitate muscle and marker identification on recordings. Markers were implanted proximally and distally in the muscle belly, as close to the origin and insertion as possible, so that a straight muscle line of action could be approximated. Due to their broad origins, multiple markers were implanted at the origins of m. latissimus dorsi (one at the scapular origin, plus one each at the cranial and caudal extremes of the fleshy vertebral origin) and m. pectoralis (one each at the cranial and caudal extremes of the sternal origin).

Veterinary tissue glue was used to secure all the bone and muscle markers and the forelimb and body was re-covered with the reflected skin and plastic wrap to prevent drying of the soft tissues during experiments.

**FIGURE 2:**
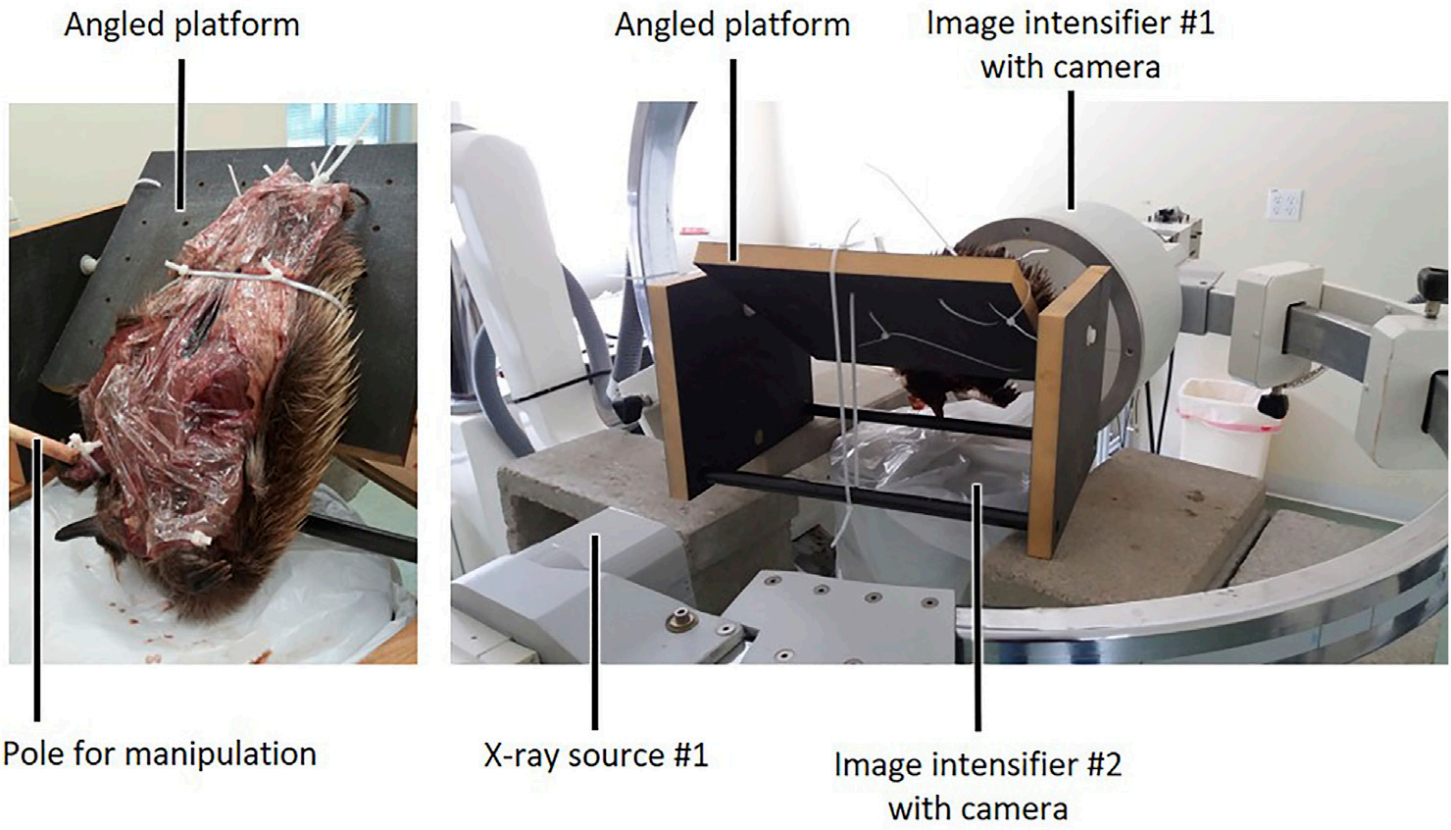
Experimental set-up for *ex vivo* passive manipulation of echidna cadaver forelimbs. After markers were placed in bones and muscles, each animal was covered with plastic wrap to prevent drying out, tied to a custom-made carbon fibre platform oriented to allow limb manipulation, and placed in the field of view of two refurbished c-arm fluoroscopes for data collection.

Each echidna cadaver was secured to an angled, custom-made carbon fibre platform, in an orientation that allowed maximal mobility of the forelimb. Typically, this was achieved by securing the hindlimbs and abdomen to the platform with cable ties through pre-drilled holes in the platform, so that the thorax and forelimbs hung over the edge (Figure 2). One forelimb was manipulated at a time using a wooden pole, so that the operator could maintain distance from the x-ray source. The pole was attached to the echidna forelimb via either a cable tie around the carpus, and/or a metal screw eye inserted into the distal humerus. Each experimental trial incorporated several cycles of differing motion: the forelimb was manipulated through maximal abduction (X+), adduction (X−), internal rotation/pronation (Y+), external rotation/supination (Y−), flexion (Z+ or Z− depending on joint), and extension (Z− or Z+), at both the glenohumeral and humeroradioulnar joints. The manipulations attempted to achieve maximum possible excursions for each DOF, through the DOF itself and combined with other motions. For example, to attempt to achieve maximum glenohumeral extension, we manipulated the limb through cycles of flexion-extension at variously abduction-adduction and internally-externally rotated positions, including approximately “neutral” (mid-point) joint positions for abduction-adduction and long-axis rotation.

Data were collected using two refurbished c-arm fluoroscopes (90–95 kV and 2–2.5 mA for 13.3 s), recorded using two high speed Photron Fastcam 1024 PCI cameras (Photron United States Inc., San Diego, CA, United States) at 60 frames per second. The relatively long recording times enabled each trial to contain several cycles of each type of limb manipulation. Between 7 and 10 trials were recorded for each side of each animal, and the best 3–5 trials (i.e., lowest error reported in XMALab, detailed below, and qualitatively judged to capture full range of motion) were selected from these for marker digitisation and analysis. In total, five trials were processed for mm. clavodeltoideus, coracobrachialis and biceps brachii, four trials for mm. pectoralis and latissimus dorsi, and three trials for m. triceps brachii superficialis longus.

### Experimental Data Processing

Trials were processed using XMALab version 1.5.1 (Knörlein et al., 2016) to calculate the transformations of the bone and muscle markers. Transformation data were filtered using a cut-off frequency 5-10x of the passively manipulated motions; values varied from trial to trial but cut-off frequencies between 3 and 8Hz were used. Filtered transformations were checked in the rigid body plot window of XMALab to ensure data were not over-filtered (i.e., plotted rigid body transformations were smoothed without changing the shape of the curve).

To calculate the rigid body transformations of the bones during each trial, the XROMM workflow requires creation and animation of specimen-specific digital bone models containing bone marker locations. To generate these models, each experimental animal was scanned at the Harvard University Center for Nanoscale Systems using a HMXST225 micro-CT system (X-Tek, Amherst, NH, United States), with the following parameters: 120 kV, 120 mA, 1s exposure, 0.25 mm copper filter. The CT projections were converted to a TIFF image stack using CT Pro 3D software (Nikon Metrology Inc., Brighton, MI, United States), then imported into Mimics version 19.0 (Materialise, Leuven, Belgium), to segment three-dimensional surface meshes of the forelimb bones and implanted bone and muscle markers.

To enable comparison between the experimental XROMM data and the SIMM model’s ROM and MMA estimates, the digital bone models of all experimental animals were aligned to the same joint coordinate system and “reference pose” as the initial model (Regnault and Pierce, 2018). The 3D bone meshes from the experimental cadavers were imported into 3ds Max 2017 (Autodesk, San Rafael, CA, United States) and the left and right-side pectoral girdle and forelimb bones of each animal were assigned anatomical joint axes and assembled into a kinematic hierarchy, as in the initial model [described in Regnault and Pierce (2018)]. The experimental animal models were then aligned via rotation around the joint axes to match the initial model specimen’s reference pose.

Although the body masses of the animals differed somewhat (2.48–3.79 kg experimental animals vs. 3.31 kg initial model specimen), much of the difference was due to body condition/fat: the bones were similarly-proportioned when overlying each other and scaling of bone meshes was not necessary. All aligned and posed experimental animal bone models were then exported to Maya 2017 (Autodesk, San Rafael, CA, United States), along with their joint axes. The rigid body transformation data from each XROMM trial was used to animate the bone meshes. The translations and rotations of the joint axes at each frame were exported as .csv files using the “exp” function of the XROMM tool shelf.

Each echidna specimen was dissected after XROMM data collection, to assess whether muscle markers had remained *in situ* during the trials, and also to collect muscle architecture data (detailed in Regnault et al. (2020). Muscles for which markers were found to have moved from the implantation site during data collection were eliminated from the analysis, and so are not reported in this study. The mm. pectoralis and latissimus dorsi muscles had broad origins, and were modelled as several lines of action. To enable valid comparison, the markers in the experimental animal specimens were implanted as close to the selected model muscle heads as possible and the position confirmed in dissection and imaging post data collection.

The muscle markers were digitised and filtered alongside bone markers in XMALab version 1.5.1 (described above). The muscle marker transformations throughout each experimental trial were then imported into Maya 2017 alongside the animated bones. The animated marker locations were used to calculate experimental MMAs, detailed below.

### Experimental MMA Calculation and Comparison With Model MMA

MMAs were calculated for each implanted muscle at each joint pose, across the full range of experimental joint ROM. This was done using a geometric method based on the mechanical definition of a moment arm being the shortest perpendicular distance from the centre of rotation to the force line of action (Sherman et al., 2013); or in the echidna’s case, the distance between the anatomical joint centre and vector running between implanted muscle markers. The 3D geometric MMAs were calculated using a custom Python script, in two steps described in detail below.

First, for each muscle-joint pairing (e.g., biceps-elbow), separate X, Y, and Z moment arms ( $\vec{r_{x}}$ , $\;\vec{r_{y}}$ , and $\vec{r_{z}}$ ) were calculated as vectors spanning the shortest perpendicular distance between two skew lines: a unit vector representing one of the three joint axes ( $\hat{x}$ , $\hat{y}$ , and $\hat{z}$ ), and a muscle vector running between the proximal and distal implanted muscle markers ( $\vec{F}$ ) (Supplementary Figure S9A). Since the shortest distance between two skew lines is always perpendicular to both lines (Supplementary Figure S9B), this is equivalent to first finding the perpendicular 3D distance between the anatomical joint centre and the muscle’s line of action, and then finding the 2D projection of that distance onto each axis’ plane of rotation. The resulting moment arm $\vec{r}$ is a vector with both direction and magnitude. A joint moment $\vec{\tau }$ may then be determined by finding the cross product of $\vec{r}$ and a force vector $\vec{F}$ , following the equation:

$$\vec{\tau }=\;\vec{r}\times \vec{F}$$ (1)

While this geometric method yields moment arms as vectors, musculoskeletal modelling programs such as SIMM use a different concept of moment arms that follows the general definition:

$$r_{\theta }=\;\frac{\tau_{\theta }}{F}$$ (2)

where $r_{\theta }$ , $\tau_{\theta }$ , and *F* are all scalars and $r_{\theta }$ is specific to each axis of rotation (Sherman et al., 2013). To convert a vector moment arm $\vec{r}$ to the scalar form $r_{\theta }$ for comparison, it is not sufficient to simply take the magnitude of $\vec{r}$ ; once $\vec{r}$ has been computed for each joint axis, its magnitude || $\vec{r}$ || must then be scaled by the fraction of the muscle vector’s magnitude that lies in the plane perpendicular to the axis, and thus capable of generating a moment about $\hat{x}$ . In other words, the scalar magnitude of the vector projection of $\vec{F}$ from axis $\hat{x}$ is divided by || $\vec{F}$ ||. The intuition for this step is straightforward: Eq. 1 contains spatial information in the form of the directions of vectors $\vec{r}$ and $\vec{F}$ , both of which are necessary to calculate a moment since $\vec{r}$ is valid for an infinite number of possible force vectors of equal magnitude (e.g., $\vec{F}\prime$ , $\vec{F}\prime \prime$ ), all intersecting with $\vec{r}$ at point *p* (Supplementary Figure S9C). This issue does not arise in the 2D case, where $\vec{F}$ is constrained to lie entirely in the same plane as $\vec{r}$ ; its entire magnitude contributes to $\tau_{\theta }$ . In 3D, it is possible for part or all of $\vec{F}$ to lie out of plane. For instance, the hypothetical $\vec{F}"$ runs antiparallel to axis $\hat{x}$ , and obviously has no capacity to generate an *x* moment, yet using || $\vec{F}"$ || rather than || $\vec{F}$ || for *F* in Eq. 2 yields the same value for $\tau_{\theta }$ , rather than the expected 0. Hence, we preserved the spatial specificity of $r_{\theta }$ by scaling || $\vec{r}$ || based on the direction of $\vec{F}$ .

To speed up analysis and increase user-friendliness by enabling quick visual inspection, the geometric moment arm script was incorporated into a Maya shelf tool. This allowed all calculations to be performed entirely within the Maya graphical interface. The tool has been made freely available as a GitHub repository, accessible at https://github.com/philsometimes/mayaMomentArms. The tool requires two sets of inputs: 1) the proximal and distal joint axes created by the jAx tool in XROMM MayaTools, and 2) a pair of animated objects representing the start and end points of a 3D muscle vector. In the present study, Maya locators animated to match implanted markers were used for the muscle points, but any arbitrary objects including “virtual” points placed directly on animated bones may be used instead.

To compare the experimentally-calculated MMAs with equivalent SIMM model estimations, MMAs from the SIMM model needed to be taken at the same poses as in each experimental trial. The rotations and translations of the joint axes at each frame of the experimental trials were used to create a SIMM motion (.mot) file with which to animate the SIMM model. An example trial is available as Supplementary Video S1.

The built-in Plot Maker function in SIMM was used to calculate muscle moment arms for comparison with the experimental MMAs. As a result, MMAs were calculated using two independent methods: the experimental MMAs are based on a geometric method of calculating MMAs described above, whilst the SIMM model uses a partial velocity method equivalent to tendon travel, using the model’s muscle paths (determined by attachment points and wrap objects). As discussed in the Introduction, the use of two different methods is valuable in validating model MMA outputs.

In addition to visualising the similarities and differences between SIMM model and experimental MMAs, we also calculated Root Mean Square Error (RSME) with the mean_squared_error function in the scikit-learn Python package (Pedregosa et al., 2011). These results can be found in Supplementary Table S2. While this provides a more quantitative perspective on the relative similarity/differences between experimental and model MMAs, RSMEs are generally employed to compare the performance of multiple models, and we caution that compared with the visual MMA plots presented here, RMSE values do not offer a more objective threshold on how well the MMA data agree.

### Muscle Architecture and Functional Signal

As part of our evaluation of the traditional musculoskeletal model workflow (e.g., Bishop et al., 2021a), we collected muscle architectural parameters: muscle belly mass and length, external tendon length, fascicle length and pennation angle, and calculation of muscle physiological cross-sectional area [methods and results are reported in Regnault et al. (2020)]. Here we wanted to better understand how robust the overall conclusions drawn from the initial model (based on MMAs) were to additional levels of modelling detail through the calculation of muscle torques. To the initial SIMM model muscle (.msl) file, we added: 1) maximum force, calculated from muscle physiological cross-sectional area (PCSA) multiplied by a muscle stress value of 0.3Nmm^−2^ (Zajac, 1989), 2) optimal fibre length (i.e., resting muscle fibre length), 3) tendon slack length, and 4) the pennation angle of inserting fibres. Four characteristic musculotendon curves (Millard et al., 2013) were also added to the SIMM .msl file: a tendon force-length curve, active and passive force-length curves, and a force-velocity curve. These are generalised curves which are not species-specific for the echidna, as those data are currently not available. Calculation of individual muscle torques around each joint for each rotational degree of freedom allowed comparison of model-calculated MMAs with muscle torques, and evaluation of how interpretations about individual muscles and whole limb function might (or might not) change with the inclusion of these additional data.

### Data Visualisation

Data in this study are presented through several visualisation methods to explore different aspects of model evaluation. The detailed methods for presented figures are described here, and summarised in the figure captions, to ensure readers can interpret figures with relevant contextual information.

**FIGURE 3:**
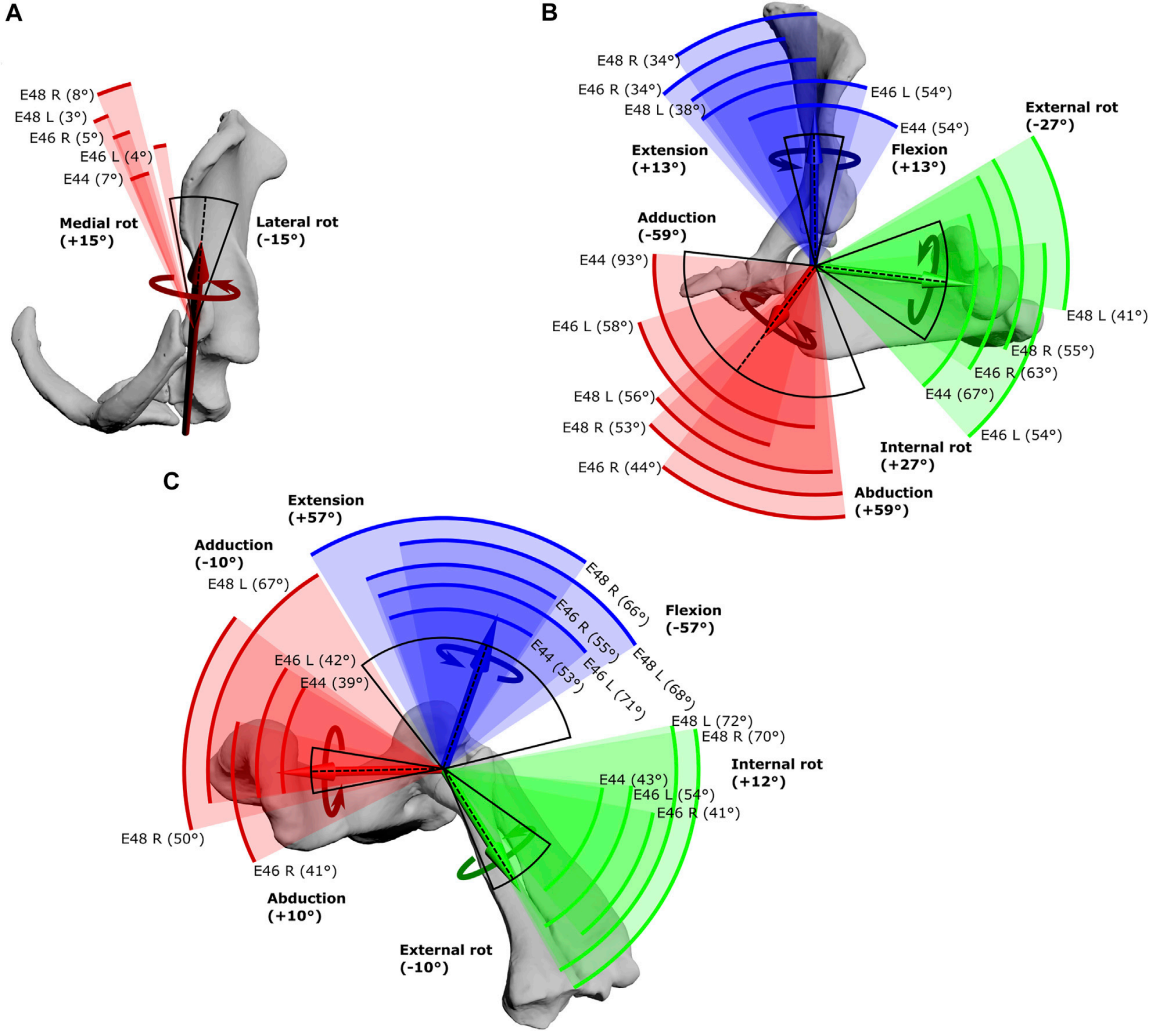
Maximal Range of Motion (ROM) at the echidna forelimb joints. **(A)** scapulocoracoid-clavicle-interclavicle joint, **(B)** glenohumeral joint, **(C)** humeroradioulnar joint. Experimentally-estimated ROMs (pooled raw data) for each echidna specimen E44, E46 and E48 (coloured arcs, total ROM in parentheses) compared with initial model-predicted ROMs based on single DOF rotations (black arc, limits in bold text) about the X (red), Y (green) and Z (blue) axes. The dotted line represents the limb’s reference position. See Table 1 for more details.

**FIGURE 4:**
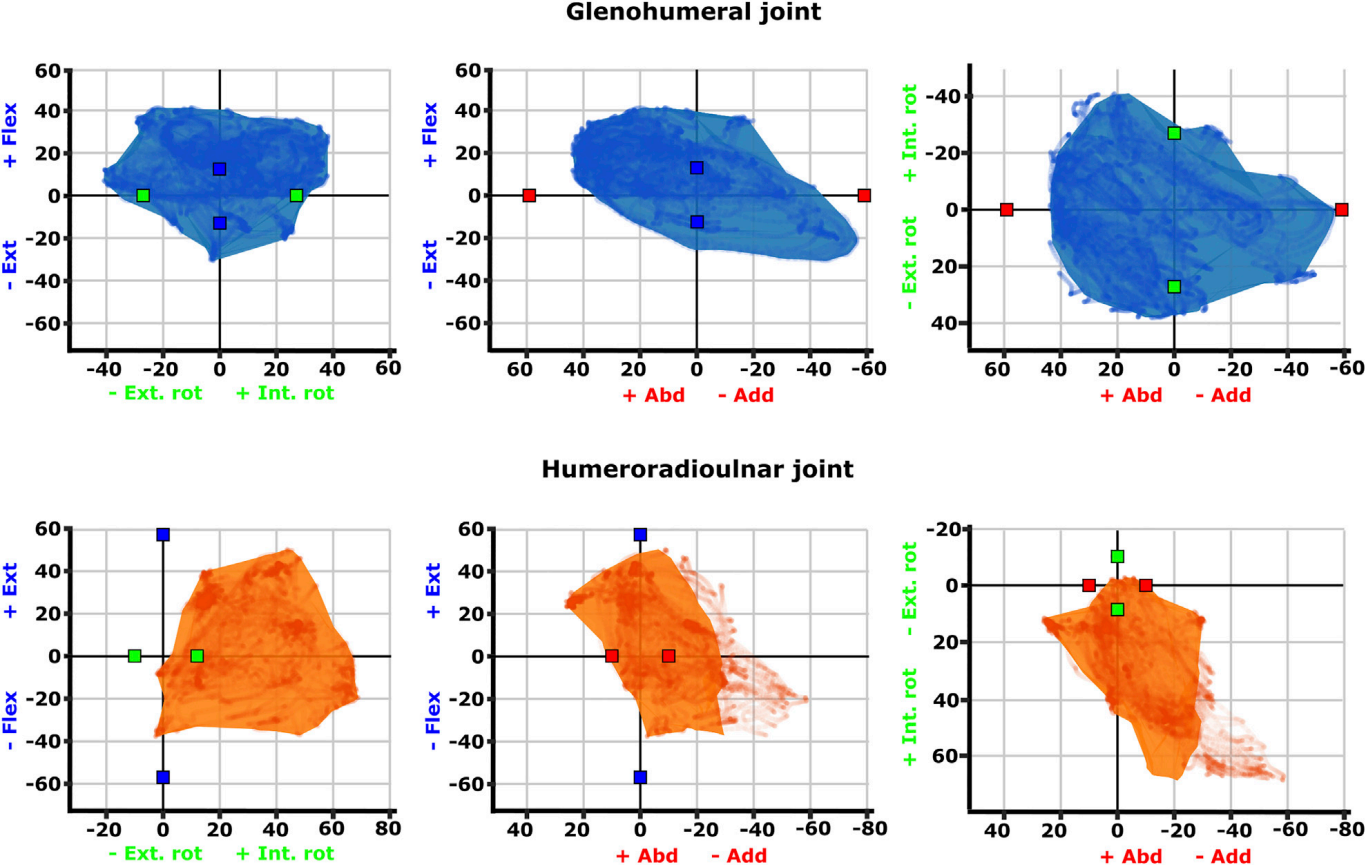
Three-dimensional (3D) joint ROM at the glenohumeral joint (top row, blue) and humeroradioulnar joint (bottom row, orange). Note that axis scale differs between plots. The 3D envelopes encompass cosine-corrected experimental ROMs wrapped in a concave hull (alpha value 20), whilst the plotted points behind in the same colour show the uncorrected experimental ROMs. The initial model-predicted maximum ROMs for single DOF rotations from the zero (reference) position have been superimposed on the 3D plots as red (X), green (Y), and blue (Z) cubes (data from Regnault and Pierce (2018)). Individual specimen trial sets are illustrated in Supplementary Figure S4.

Experimental joint ROMs are presented as rotational ranges in both raw (uncorrected) Euler space (Figure 3) as well as cosine-corrected Euler space (Figure 4). Joint ROMs are often presented as maximum excursions per rotational DOF or plotted in uncorrected Euler space, but these visualisations can preclude comparisons amongst joints with different joint coordinate systems and distort comparisons between joint space volumes (for example, equally-different poses not being depicted equally far apart) (Manafzadeh and Gatesy, 2020). Cosine-correction is a method that has been recently developed and applied to 3D depictions of model pose-space, addressing these issues (Manafzadeh and Gatesy, 2020). Here, we have chosen to present both types of visualisation. For the uncorrected visualisation (Figure 3), the maximal joint angles achieved across each experimental animal’s trials were pooled (i.e., the largest joint angle for each motion and joint taken for each animal) to be directly compared to the initial SIMM model-estimated maximal ROMs (Regnault and Pierce, 2018). For the cosine-corrected ROMs (Figure 4), all joint transformations (i.e., pose per frame of experimental trial) were imported into Python (Van Rossum and Drake, 1995) and cosine-correction performed on the axis of greatest variation (X-axis). The plotted points of cosine-corrected joint transformations were then wrapped in a concave hull (or alpha shape) to visualise ROM as a 3D “envelope” with an alpha threshold of 20. Uncorrected point clouds are presented alongside the hull envelopes to enable comparison with the initial SIMM model maximal ROM estimates, since both use the same joint coordinate system.

**FIGURE 5:**
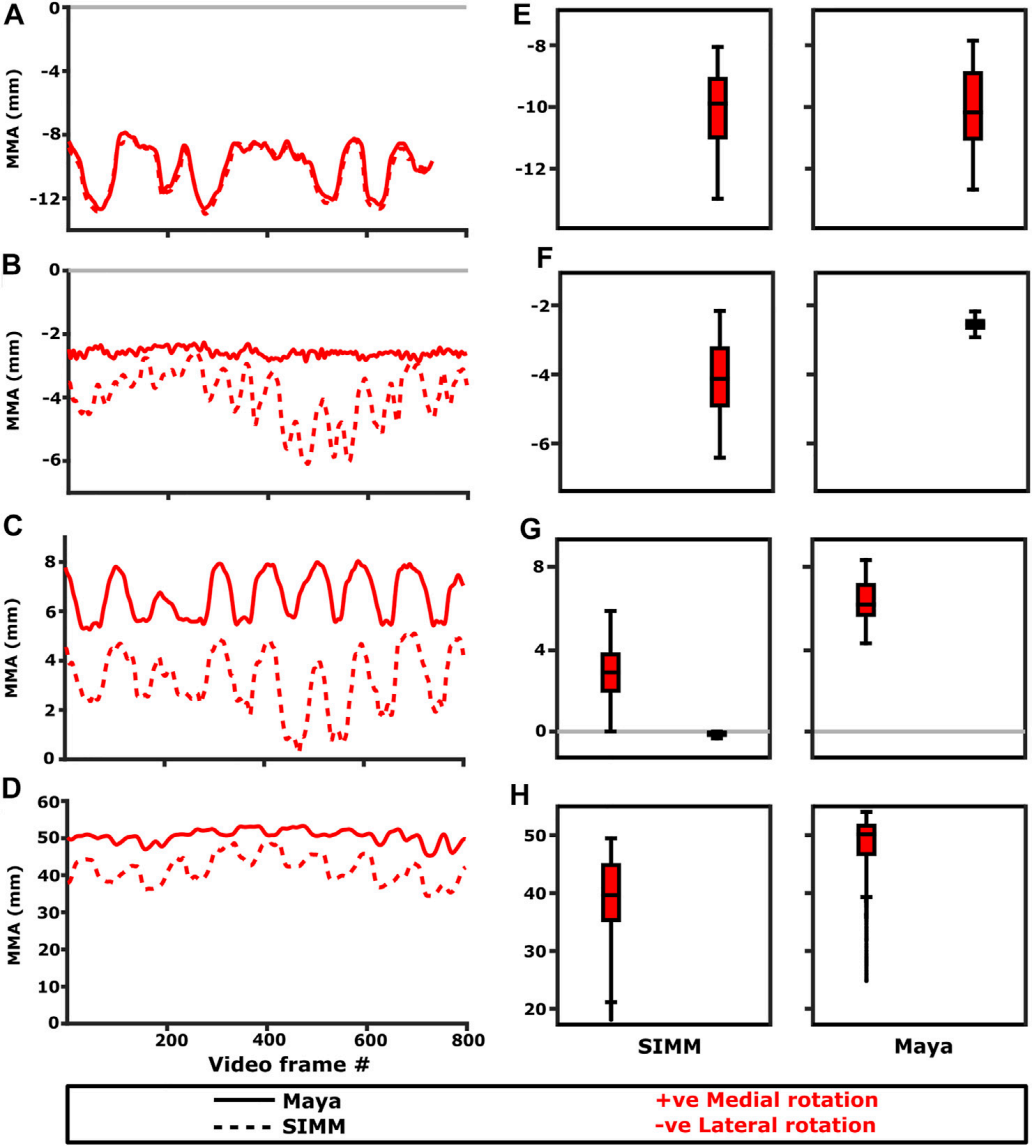
Representative trials (left) and frequency distributions of MMAs for all kinematic trials (right) for muscles crossing the scapulocoracoid-clavicle-interclavicle joint. Representative trials for: **(A)** m. clavodeltoideus, **(B)** m. pectoralis cranial origin, **(C)** m. pectoralis caudal origin, **(D)** m. latissimus dorsi vertebral origin. The boxplots (**E–H)** show the distribution and median values of MMAs for all trials: **(E)** m. clavodeltoideus, **(F)** m. pectoralis cranial origin, **(G)** m. pectoralis caudal origin, **(H)** m. latissimus dorsi vertebral origin. Positive and negative MMAs are plotted as separate boxplots for each DOF. SIMM = model-predicted MMAs based on partial velocity; Maya = experimentally-calculated MMAs based on the geometric method.

**FIGURE 6:**
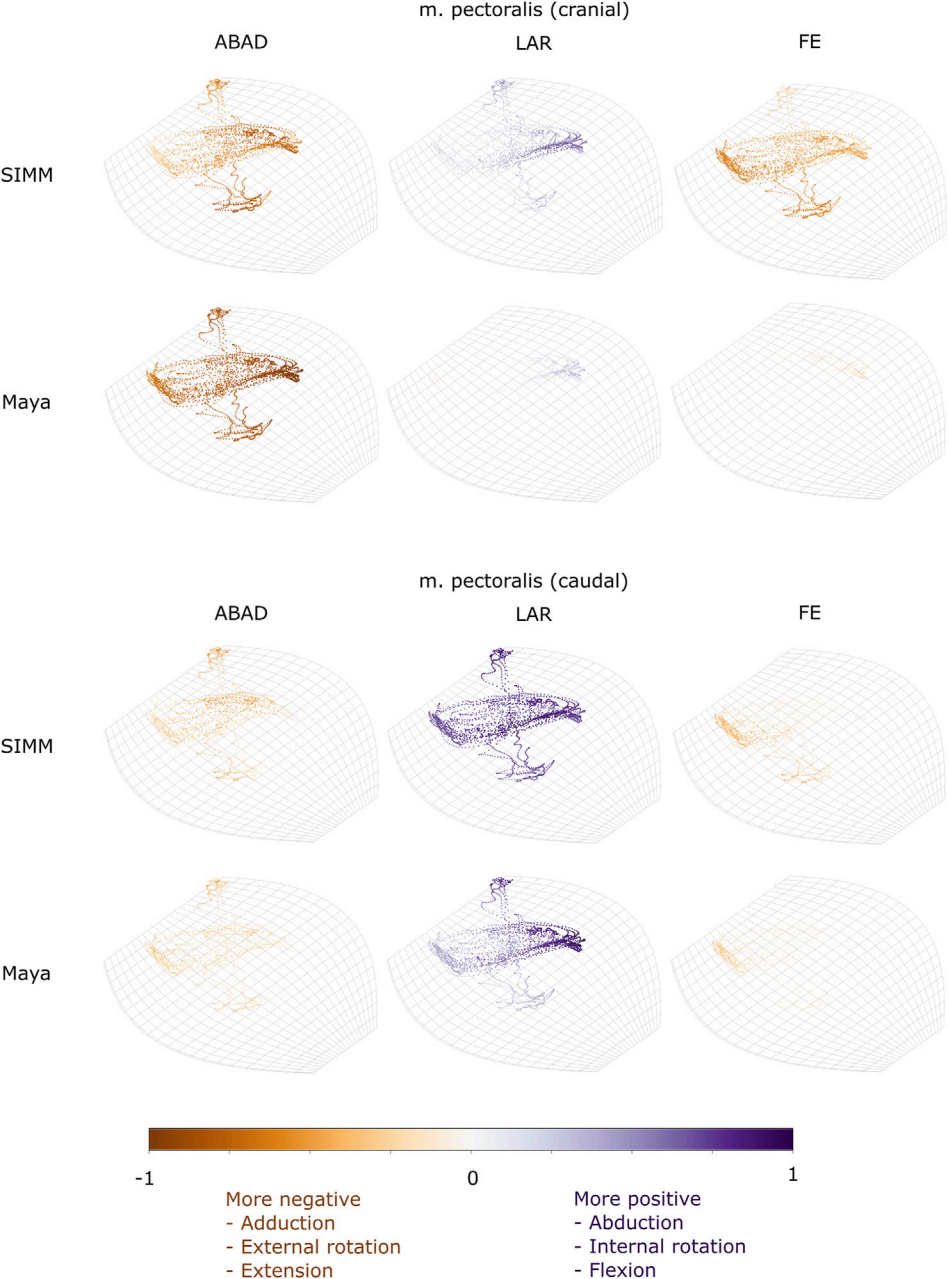
Three-dimensional (3D) MMAs of m. pectoralis at the glenohumeral joint, plotted in cosine-corrected ROM space for the SIMM model (using partial velocity) and Maya experimental data (using the geometric method). MMA sign is indicated by point colour (positive values are purple, negative values are orange) with colour intensity scaled to relative MMA magnitude as described in the Methods. ABAD = abduction-adduction; LAR = long-axis rotation (internal-external rotation); FE = flexion-extension.

**FIGURE 7:**
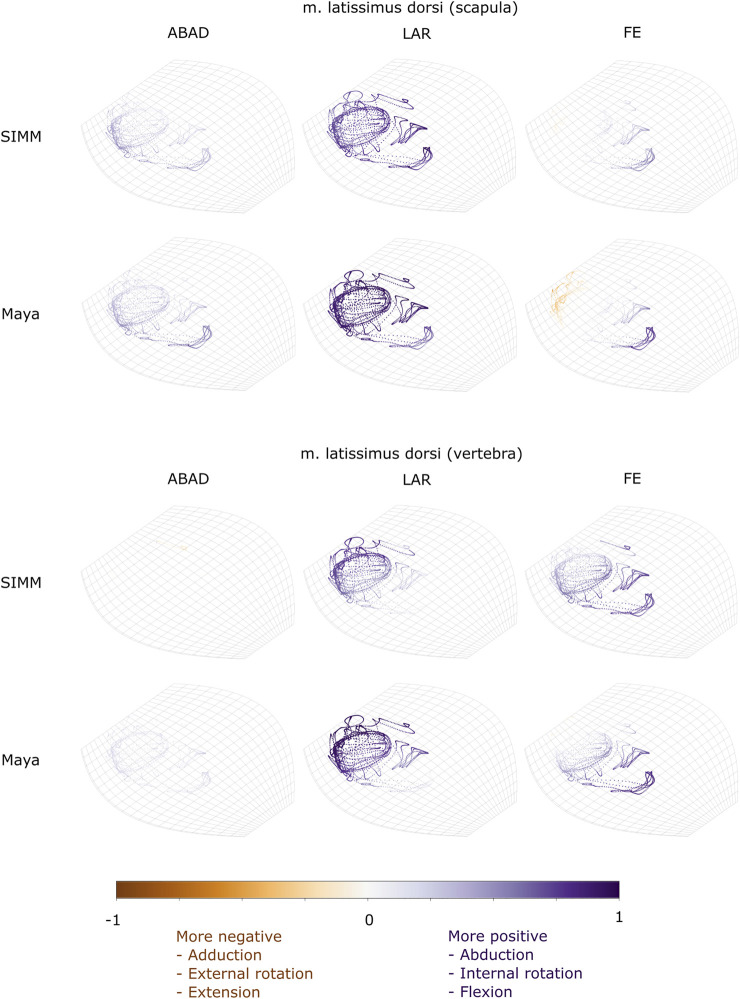
Three-dimensional (3D) MMAs of m. latissimus dorsi at the glenohumeral joint, plotted in cosine-corrected ROM space for the SIMM model (using partial velocity) and Maya experimental data (using the geometric method). MMA sign is indicated by point colour (positive values are purple, negative values are orange) with colour intensity scaled to relative MMA magnitude as described in the Methods. ABAD = abduction-adduction; LAR = long-axis rotation (internal-external rotation); FE = flexion-extension.

**FIGURE 8:**
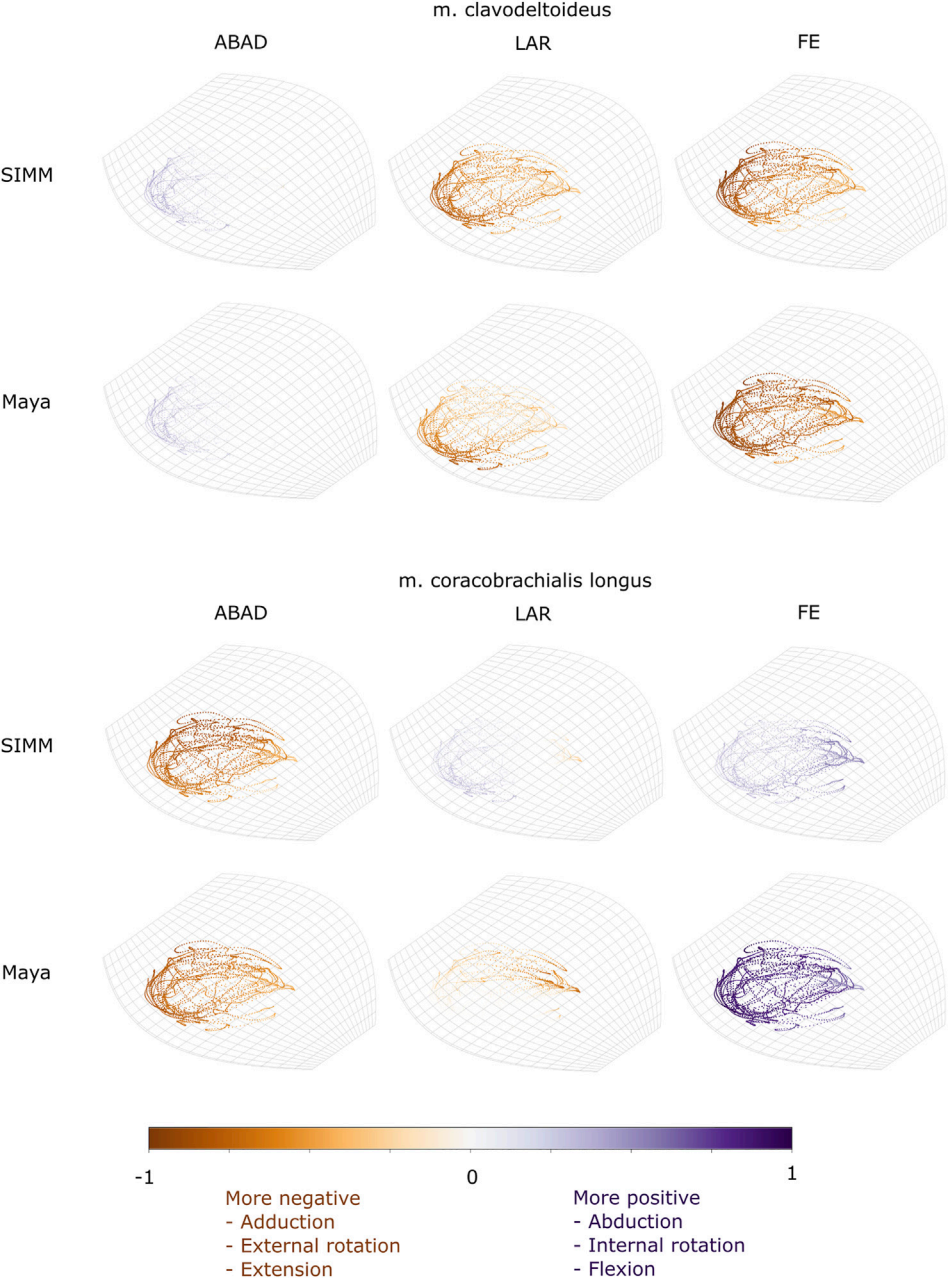
Three-dimensional (3D) MMAs of m. clavodeltoideus and m. coracobrachialis longus at the glenohumeral joint, plotted in cosine-corrected ROM space for the SIMM model (using partial velocity) and Maya experimental data (using the geometric method). MMA sign is indicated by point colour (positive values are purple, negative values are orange) with colour intensity scaled to relative MMA magnitude as described in the Methods. ABAD = abduction-adduction; LAR = long-axis rotation (internal-external rotation); FE = flexion-extension.

**FIGURE 9:**
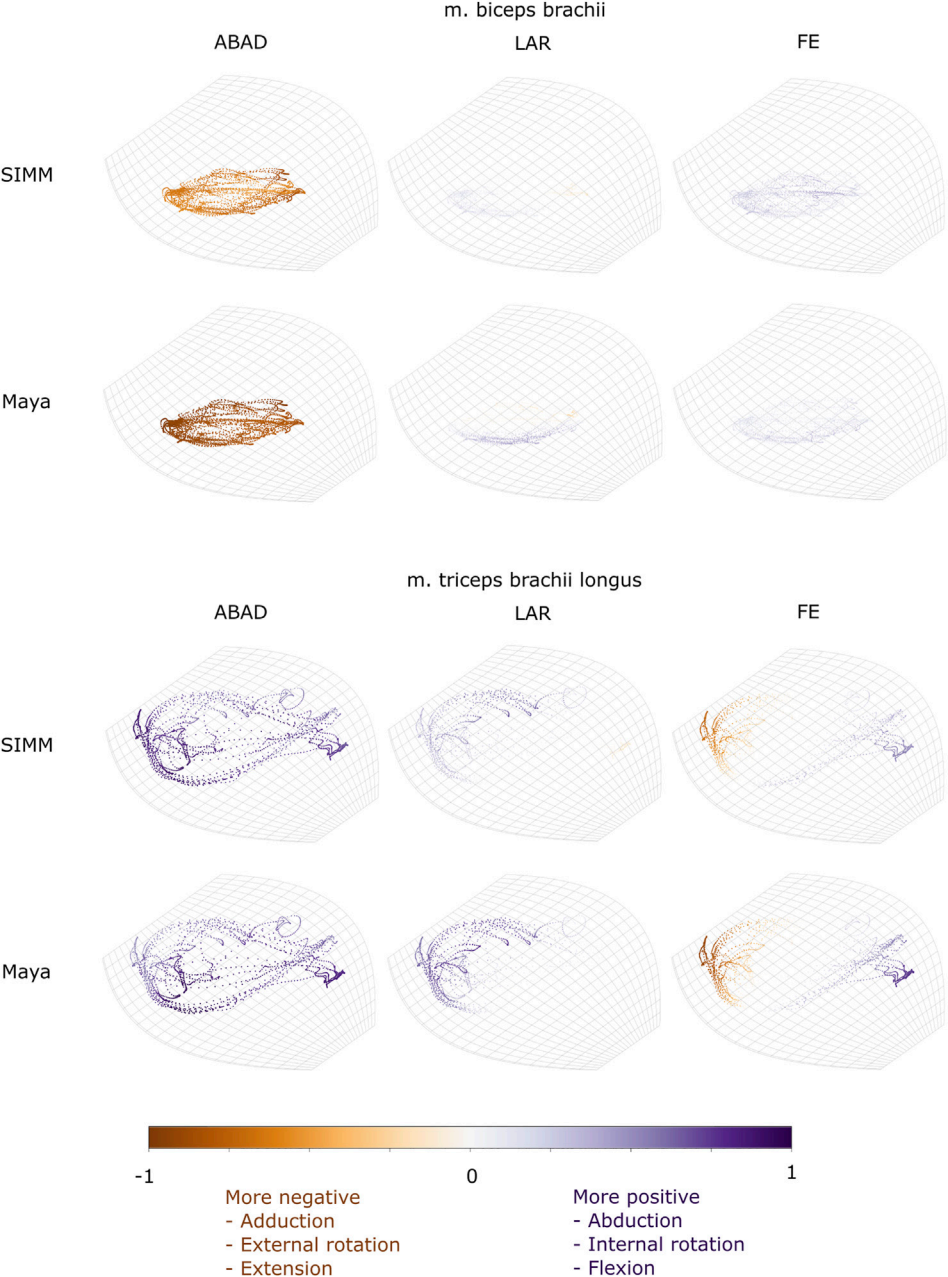
Three-dimensional (3D) MMAs of m. biceps brachii and m. triceps brachii at the glenohumeral joint, plotted in cosine-corrected ROM space for the SIMM model (using partial velocity) and Maya experimental data (using the geometric method). MMA sign is indicated by point colour (positive values are purple, negative values are orange) with colour intensity scaled to relative MMA magnitude as described in the Methods. ABAD = abduction-adduction; LAR = long-axis rotation (internal-external rotation); FE = flexion-extension. Plot for these muscles MMAs at the humeroradioulnar joint can be found in Supplementary Figure S5.

**FIGURE 10:**
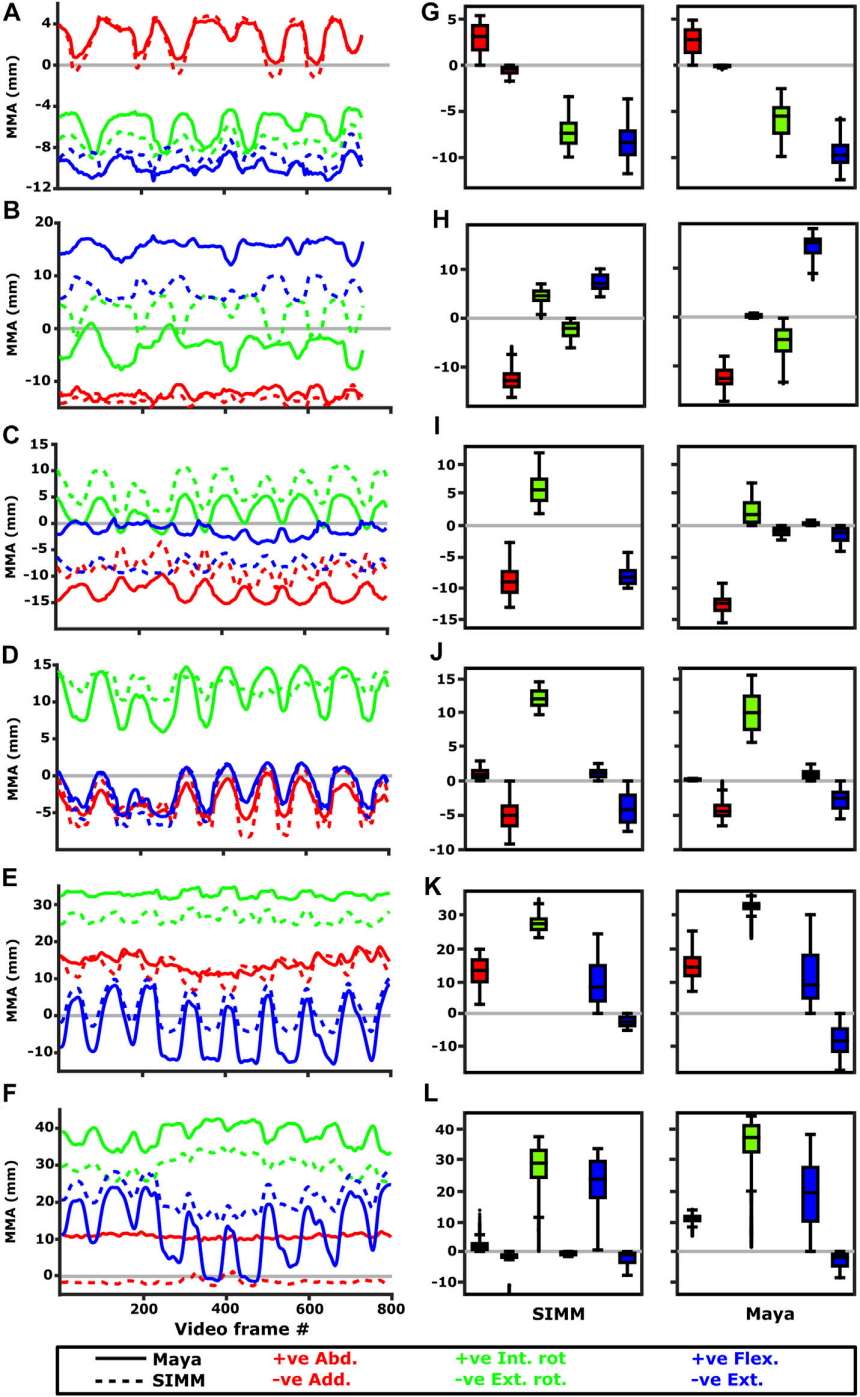
Representative kinematic trials (left) and frequency distributions of MMAs for all kinematic trials (right) for muscles crossing the glenohumeral joint. Representative trials for **(A)** m. clavodeltoideus, **(B)** m. coracobrachialis, **(C)** m. pectoralis cranial origin, **(D)** m. pectoralis caudal origin, **(E)** m. latissimus dorsi scapular origin, **(F)** m. latissimus dorsi vertebral origin. The boxplots **(G–L)** show the distribution and median values of MMAs for all trials: **(G)** m. clavodeltoideus, **(H)** m. coracobrachialis, **(I)** m. pectoralis cranial origin, **(J)** m. pectoralis caudal origin, **(K)** m. latissimus dorsi scapular origin, **(L)** m. latissimus dorsi vertebral origin. Positive and negative MMAs are plotted as separate boxplots for each DOF. SIMM = model-predicted MMAs based on partial velocity; Maya = experimentally-calculated MMAs based on the geometric method.

**FIGURE 11:**
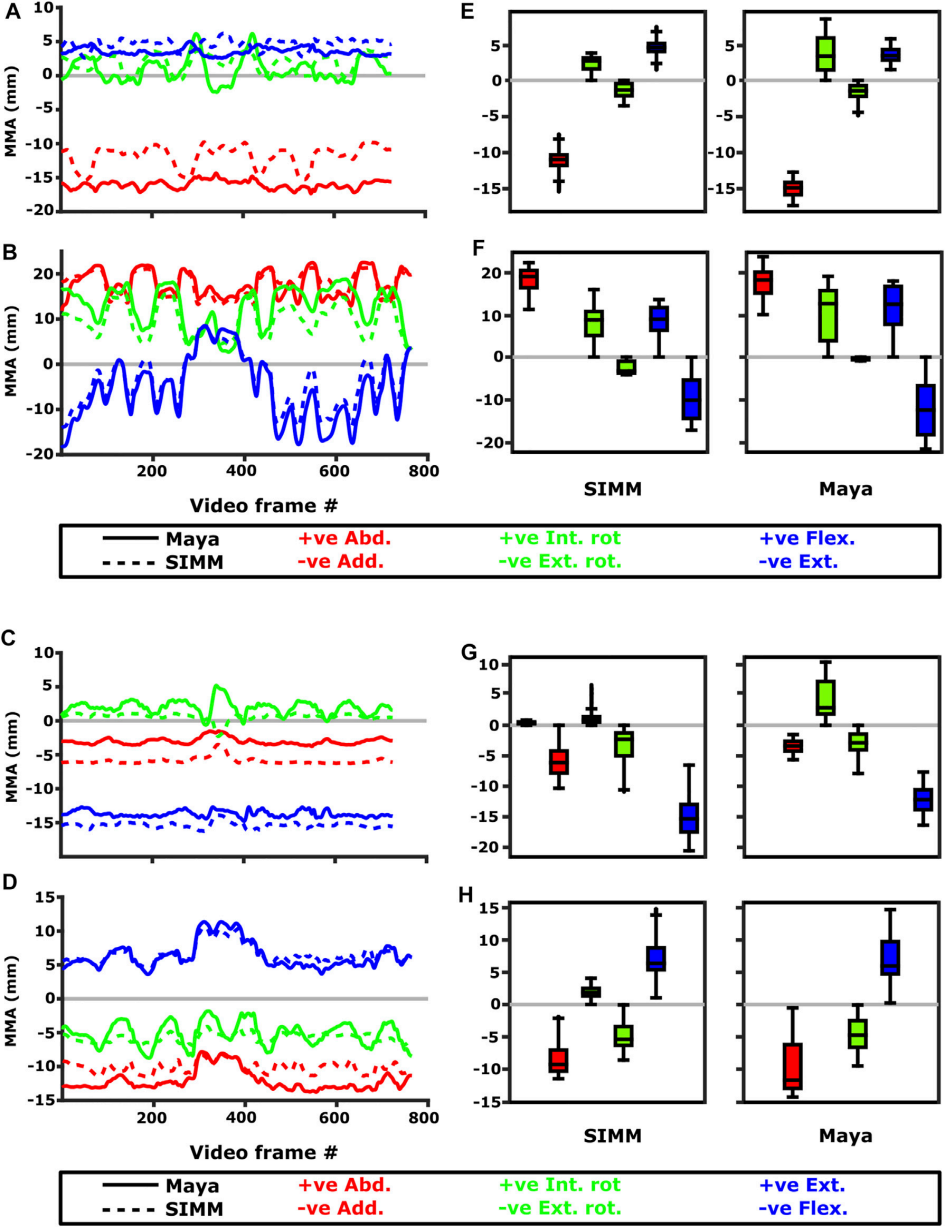
Representative kinematic trials (left) and frequency distributions of MMAs for all kinematic trials (right) crossing the glenohumeral (top) and humeroradioulnar joints (bottom). Representative trials for **(A)** m. biceps brachii at glenohumeral joint, **(B)** m. triceps brachii at glenohumeral joint, **(C)** m. biceps brachii at humeroradioulnar joint, **(D)** m. triceps brachii at humeroradioulnar joint. The boxplots **(E–H)** show the distribution and median values of MMAs for all trials: **(E)** m. biceps brachii at glenohumeral joint, **(F)** m. triceps brachii at glenohumeral joint, **(G)** m. biceps brachii at humeroradioulnar joint, **(H)** m. triceps brachii at humeroradioulnar joint. Positive and negative MMAs are plotted as separate boxplots for each DOF. SIMM = model-predicted MMAs based on partial velocity; Maya = experimentally-calculated MMAs based on the geometric method.

Experimentally-calculated 3D MMAs vs. SIMM model 3D MMAs for each joint pose are also presented as plots in cosine-corrected Euler space (Figures 6–9, Supplementary Figure S5). For these plots, the colour indicates whether the MMA is positive (purple) or negative (orange), with colour intensity indicating relative magnitude (normalised to maximum absolute value for each muscle, inclusive of the SIMM and experimental estimates). The sign (positive or negative) denotes the direction of the torque, according to the joint coordinate system. For example, a positive MMA at the glenohumeral X-axis would cause an abduction moment (torque) whilst a negative MMA would cause an adduction moment. The 3D MMA plots for the glenohumeral joint are provided in the main paper, while those for the humeroradioulnar joint can be found in the Supplementary Figure S5. Absolute (non-normalised) MMA magnitudes are also presented more traditionally for each rotational axis (Figures 5, 10, 11), as representative kinematic trials for each muscle and as frequency distribution boxplots to assess magnitudes and rank orders.

Finally, individual and summed MMAs and torques were determined at the glenohumeral joint and compared to evaluate inferred functions and muscle roles using the initial SIMM model’s ROM (single-axis DOF rotations as is typical of traditional musculoskeletal models). To avoid over-representation of muscles with multiple modelled heads (e.g., mm. biceps brachii, m. latissimus dorsi), mean values were determined for these muscles before calculation of the summed MMAs [as described in Regnault and Pierce (2018)]. Comparisons between individual MMAs and their corresponding torques are presented in the Supplementary Figures S1–S3, S6–S8.

## Results

### Muscle Pathways

Several muscle attachment points on the initial SIMM model were adjusted following diceCT and digital segmentation, to better reflect specimen-specific anatomy (see Supplementary Table S1). Many of the muscles that were adjusted were done so due to small or narrow attachment areas differing between Gambaryan et al. (2015), on which the initial model was based, and our specimen-specific attachment areas, but with negligible effects on the initial model-predicted MMAs (Supplementary Figures S1–S3). However, a few muscle attachments genuinely appeared to differ from Gambaryan et al. (2015), and thus more significant adjustments were deemed necessary, as noted below.

One muscle was m. pectoralis. The cranial-most extent of this muscle’s origin (“part 1” in the initial model) was far more cranial in the literature reconstruction than in our contrast-stained and segmented specimen. Gambaryan et al. (2015) observed this muscle to originate from the interclavicle cranially, but our specimen’s cranial-most border was the first sternal element (sometimes called the presternum; shown in Figure 1B, 2B of Regnault et al., 2020). Moving parts 1 and 2 of m. pectoralis in the model (representing the cranial-most and middle body of the muscle origins) affected the resultant estimated MMAs for these parts of m. pectoralis (Supplementary Table S1, Supplementary Figures S1, S2H). At the scapulocoracoid-clavicle-interclavicle joint, the moment arms for parts 1 and 2 of m. pectoralis become less negative (Supplementary Figure S1): part 1 thus is interpreted to produce a much weaker lateral scapulocoracoid movement, whilst part 2’s moment arms now cross zero with a negative slope, suggesting it may act as an intrinsic joint stabiliser (albeit weakly). Similarly, at the glenohumeral joint, m. pectoralis part 1 (cranial part) now has positive moment arm values in long-axis rotation; in other words, acting to internally rotate/pronate the humerus, similar to the other parts of m. pectoralis (Supplementary Figure S2H). Overall, at the glenohumeral joint, the MMAs for the parts of m. pectoralis appear more similar to one another compared with the initial model, indicating these muscle parts would produce similar actions when contracting.

The other muscle with greater discrepancy was m. biceps brachii longus. In the initial model, this head originated from the epicoracoid (following Gambaryan et al., 2015). In our diceCT specimen (and accompanying dissections of other specimens; Regnault et al., 2020), we confirmed an epicoracoid attachment, but note that the bulk of the muscle originated on the coracoid (alongside m. biceps brevis). Altering its origin from the more modest epicoracoid attachment site to the larger scapulocoracoid site resulted in m. biceps longus no longer crossing the scapulocoracoid-clavicle-interclavicle joint in the model, and so the small moment arm at this joint is removed (Supplementary Figure S1).

The muscle paths of mm. biceps brachii brevis and longus were also adjusted to better follow the centroid of 3D muscle meshes. Subsequent to the muscle origin and path adjustments in the revised model, the flexion-extension moment arms of mm. biceps at the glenohumeral joint changed signs, altering their interpreted action from external rotators/supinators to internal rotators/pronators. The flexion-extension moment arm for m. biceps brevis also switched from negative to positive, changing its interpreted action at the glenohumeral joint from an extensor to flexor. In a similar fashion, adjustment of muscle paths for parts of mm. deltoid resulted in the glenohumeral abduction moment arm for m. clavodeltoideus to switch sign at greater angles of adduction (i.e., becomes an adductor), and the mixed flexor-extensor moment arms of m. acromiodeltoideus to become wholly negative (i.e., only extensor).

### Joint Range of Motion

The scapulocoracoid-clavicle-interclavicle joint possesses only a single rotational degree of freedom (Figure 3A). Pooled experimental data from all echidna trials encompass a ROM totalling 17° of motion around this rotational axis (Table 1). However, individual ranges for each animal are more limited (Figure 3A), and so the experimentally-estimated ROMs are much less than the initial model-predicted ROM of 30°.

**TABLE 1:** Raw, un-cosine-corrected values of experimentally-estimated rotations and translations at the echidna forelimb joints. Total joint ROM is in parentheses.

Echidna #	E44	E46		E48		Total possible
Side: Left (L), Right (R)	L	L	R	L	R	
Scapulocoracoid-clavicle-interclavicle medial (X+) lateral (X−) rotation angle (°)	21 to 28 (7)	14 to 18 (4)	23 to 28 (5)	27 to 31 (3)	20 to 28 (8)	14 to 31 (17)
Glenohumeral abduction (X+) adduction (X−) angle (°)	−57 to 37 (93)	−35 to 23 (58)	0 to 44 (44)	−13 to 42 (56)	−9 to 44 (53)	−57 to 44 (100)
Glenohumeral internal (Y+) external (Y−) long-axis rotation angle (°)	−41 to 26 (67)	−41 to 13 (54)	−27 to 36 (63)	−3 to 38 (41)	−17 to 38 (55)	−41 to 38 (79)
Glenohumeral flexion (Z+) extension (Z−) angle (°)	−31 to 24 (54)	−17 to 37 (54)	7 to 41 (34)	−1 to 36 (38)	−1 to 33 (34)	−31 to 41 (72)
Glenohumeral cranial (X+) caudal (X−) translation (mm)	−5.9 to 4.5 (10.4)	−4.5 to 4.3 (8.8)	−5.4 to 1.7 (7.1)	−2.6 to 2.7 (5.3)	−2.5 to 3.6 (6.1)	−5.9 to 4.5 (10.4)
Glenohumeral proximal (Y−) distal (Y+) translation (mm)	−0.4 to 2.7 (3.1)	−0.9 to 2.4 (3.3)	−0.9 to 2.6 (3.5)	0.1 to 2.6 (2.5)	0.5 to 2.5 (2.0)	−0.9 to 2.7 (3.6)
Glenohumeral dorsal (Z+) ventral (Z−) translation (mm)	−3.8 to 0.1 (3.9)	−3.2 to 0.4 (2.8)	0.6 to 3 (2.4)	−2.9 to 0.4 (2.5)	0.3 to 2.7 (2.4)	−3.8 to 3.0 (6.8)
Humeroradioulnar abduction (X+) adduction (X−) angle (°)	−31 to 8 (39)	−34 to 8 (42)	−14 to 26 (41)	−59 to 8 (67)	−38 to 13 (50)	−59 to 26 (85)
Humeroradioulnar internal (Y+) external (Y−) long-axis rotation angle (°)	7 to 50 (43)	1 to 54 (54)	8 to 48 (41)	−3 to 69 (72)	−2 to 67 (70)	−3 to 69 (72)
Humeroradioulnar flexion (Z−) extension (Z+) angle (°)	−15 to 38 (53)	−32 to 39 (71)	−15 to 40 (55)	−38 to 30 (68)	−16 to 50 (66)	−38 to 50 (88)
Humeroradioulnar cranial (X+) caudal (X−) translation (mm)	−1.5 to 1.6 (3.1)	−1.0 to 1.8 (2.8)	−1.2 to 1.0 (2.2)	−2.4 to 0.5 (2.9)	−1.7 to 1.3 (3.0)	−2.4 to 1.8 (4.2)
Humeroradioulnar proximal (Y−) distal (Y+) translation (mm)	−1.2 to 0.2 (1.4)	−1.9 to 0.4 (2.3)	−2.0 to 1.6 (3.6)	−1.9 to 1.5 (3.4)	−1.9 to 0.4 (2.3)	−2.0 to 1.6 (3.6)
Humeroradioulnar pre (Z−) postaxial (Z +) translation (mm)	−2.3 to 1.5 (3.8)	−1.9 to 2.6 (4.5)	−0.9 to 3.0 (3.9)	−3.6 to 1.5 (5.1)	−1.3 to 4.0 (5.3)	−3.6 to 4.0 (7.6)

Conversely, at the glenohumeral and humeroradioulnar joints, experimental ROMs approached and/or exceeded the model-predicted ranges (Figures 3B,C and Table 1). At the glenohumeral joint, abduction-adduction was greatest across all specimens and trials, totalling 101°, followed by long-axis rotation at 79° and flexion-extension at 72°. This is a similar pattern as predicted by the initial model using single-axis DOF rotations: total ROMs were 118° in abduction-adduction, 54° in long-axis rotation and 26° in flexion-extension (Regnault and Pierce, 2018). Of particular note here, flexion-extension ROM was much greater at the glenohumeral joint during the experimental trials. Experimental data also showed that translations at the joint surfaces occurred, the greatest being 10.4 mm along the X (craniocaudal) axis, with less translation along the other axes (Table 1).

At the humeroradioulnar joint, experimental ROM was similar around each of the rotational degrees of freedom (Figure 3C): flexion-extension was greatest, totalling 88°, followed by abduction-adduction at 85°, and then long-axis rotation at 72°. This contrasts with the initial model that predicted a predominance of flexion-extension (114° total) over the other two movements (20° and 22° for long-axis rotation and abduction-adduction respectively). Interestingly, the experimental data show long-axis rotation to consist almost completely of internal rotation (pronation) from the reference pose, with internal rotation accounting for 69° of the total 72° ROM. Joint translations were also evident at the humeroradioulnar joint, particularly along the Z (mediolateral) axis, recorded at up to 5.1 mm (Table 1).

Visualisation of the 3D glenohumeral and humeroradioulnar ROMs (Figure 4) shows the envelope of motion elicited experimentally, inclusive of simultaneous rotations and translations at the joints (up to 6 DOF). At the glenohumeral joint, these 3D plots (volume = 163,942 cubic degrees) show the initial model-predicted maximum ROMs using a single rotational DOF were very close to the experimental ROMs in abduction-adduction and long-axis rotation. However, flexion-extension ROM in the initial model greatly underpredicts possible motion in this region of pose space. It can be seen from the 3D plot in Figure 4 that the increased ROM achieved in glenohumeral flexion-extension experimental data is not wholly due to combined rotations, but more likely due to translations at the articular surface (especially along the craniocaudal axis, given the recorded translations and the elongated morphology of the echidna glenoid). In contrast, other motions, such as maximal humeral adduction, are achieved only alongside rotations in other axes (in this case, with concomitant maximum extension of the humerus; Figure 4). Supplementary Figure S4 illustrates how each specimen/side contributes to the total pooled ROM.

The 3D envelope of experimental ROM at the humeroradioulnar joint (volume = 79,303 cubic degrees) more clearly shows the limitations of the single rotational DOF method in predicting ROM. The experimental ROM envelope only occupies some regions within the model-predicted limits. In particular, far more internal rotation (pronation) occurs than the initial model predicted, with peak internal rotation occurring concomitant with near-maximal joint adduction (Figure 4). Further, maximal humeroradioulnar joint abduction occurs at high extension angles and adduction at high flexion angles. Experimental flexion-extension ROM falls within model-predicted limits, though maximal extension only co-occurs with humeral internal rotation. As above, individual specimen trial sets are illustrated in Supplementary Figure S4. These results highlight the importance of interactions between DOF in determining possible joint motions – a traditional single DOF approach in the echidna, as shown here, can miss joint poses that are achievable and include those which are not achievable in the real animal, but the degree of mismatch appears to be joint and direction dependent.

### Muscle Moment Arms

Six muscles crossing the scapulocorcacoid-clavicle-interclavicle, glenohumeral and humeroradioulnar joints were successfully implanted with markers. These muscles were: m. clavodeltoideus, m. coracobrachialis (pars longus), m. triceps brachii (pars superficialis longus), m. biceps brachii, m. pectoralis, and m. latissimus dorsi. To evaluate MMAs, experimentally-estimated muscle moment arms using the geometric method were compared to those predicted by the updated SIMM model animated with the experimental trial kinematics.

#### M. Clavodeltoideus

The m. clavodeltoideus crosses both the scapulocoracoid-clavicle-interclavicle and glenohumeral joints. At both joints, the experimentally-estimated MMAs and SIMM model-predicted MMAs agreed well. The MMAs were consistent in sign, magnitude, and rank order. At the scapulocoracoid-clavicle-interclavicle joint, both SIMM model and experimental MMAs show m. clavodeltoideus to laterally rotate the scapulocoracoid, assuming an unloaded limb (Figures 5A,E). At the glenohumeral joint, both consistently show this muscle (in order of largest to smallest MMA) to extend (i.e., protract), externally rotate (i.e., supinate), abduct, and minimally adduct the humerus (Figure 8 and Figures 10A,G).

#### M. Coracobrachialis (Pars Longus)

The m. coracobrachialis (pars longus) crosses the glenohumeral joint only. The MMAs in abduction-adduction agreed well between the experimental estimates and model predictions, being consistent in sign (i.e., adduction) and magnitude (Figure 8), as well as pattern of MMA peaks and troughs (Figure 10B). The MMAs for flexion-extension agreed in sign (i.e., flexion), but deviated in magnitude, and MMAs for long-axis rotation did not agree well in either sign or magnitude (Figure 8). The rank order was somewhat consistent (Figure 10H), with abduction-adduction MMAs generally largest (although the large MMAs for flexion-extension equalled abduction-adduction in experimental trials), followed by flexion-extension and then long-axis rotation.

#### M. Biceps Brachii

Both long and short heads of m. biceps brachii cross the glenohumeral and humeroradioulnar joints. These two heads could not be distinguished separately at marker implantation, and so the SIMM modelled m. biceps brachii short head (pars brevis) was chosen to compare with the experimentally-estimated MMAs of m. biceps brachii. The modelled head of m. biceps longus was not chosen due to the large artefactual deviations in the muscle head’s geometry when animated with the experimental trial kinematics, resulting from idiosyncratic interaction with its wrap object. The modelled m. biceps brevis showed similar MMA values to m. biceps longus (Regnault and Pierce, 2018), without such artefactual wrap object interactions.

At the glenohumeral joint, the MMAs were approximately consistent in sign and magnitude (Figure 9), though more negative adduction and internal rotation was evident in the experimental MMAs. In terms of rank order (Figures 11A,E), both the experimentally-estimated and model-predicted MMAs were largest in adduction. The comparatively smaller MMAs for flexion and internal/external rotation were less consistent: in the SIMM model, flexion-extension generally exceeded long-axis rotation whereas the experimental estimates overlapped in value. The patterns in MMA peaks and troughs during motion were not always consistent between model and experimental data (Figure 11A).

At the humeroradioulnar joint, there was also some agreement. The signs of MMAs were consistent, although long-axis rotation MMAs did fluctuate around zero and so were occasionally inconsistent in sign (Supplementary Figure S5). The magnitudes were generally consistent, though the SIMM model exhibited generally larger abduction and flexion MMAs (Supplementary Figure S5). In terms of rank order (Figures 11C,G), flexion MMAs were consistently the largest. Abduction-adduction and long-axis rotation MMAs were smaller, but their rank order sometimes varied inconsistently between the model and experimental MMAs, depending on the trial kinematics. Like the glenohumeral joint, during parts of some trials there were inconsistent patterns in MMA peak and troughs between model and experimental data (Figure 11C).

#### M. Triceps Brachii (Pars Superficialis Longus)

The long superficial head of m. triceps brachii crosses both the glenohumeral and humeroradioulnar joints. At both the joints, the experimentally-estimated MMAs and model-predicted MMAs agreed well. At the glenohumeral joint, the MMAs were consistent in sign and magnitude (Figure 9). The kinematic trial peaks/troughs and rank order (Figures 11B,F) were also consistent. Both the experimental and SIMM model MMAs show this muscle to adduct the humerus, with similar MMAs overall in flexion, extension and internal rotation, plus minimal external rotation. At the humeroradioulnar joint, results were similar (Supplementary Figure S5 and Figures 11D,H); the experimental and SIMM model MMAs show m. triceps brachii to extend and adduct the antebrachium, with lower MMAs for external rotation. The model also predicts capability for internal rotation, not shown by the experimental MMAs.

#### M. Pectoralis

The m. pectoralis crosses both the scapulocoracoid-clavicle-interclavicle and glenohumeral joints. To capture this muscle’s broad origin across the sternum, it was modelled with three origins (cranial, mid, and caudal). Only the cranial-most and caudal-most areas of m. pectoralis’ origin were implanted experimentally. Therefore, in our comparisons, we compare the cranial and caudal origin points of m. pectoralis in the SIMM model and experimental data.

At the scapulocoracoid-clavicle-interclavicle joint, MMAs were generally consistent in sign for both the cranial-most and caudal-most origins (Figures 5B,C); the cranial origin is interpreted as drawing the scapulocoracoid laterally (due to negative MMAs) whilst the caudal origin is interpreted as drawing the scapulocoracoid medially (due to positive MMAs). However, the magnitudes of model-predicted MMAs were not in particularly close agreement with experimental estimates. The model-predicted MMAs of the cranial origin were generally larger, with peaks and troughs that did not correspond well with the pattern of the experimentally-estimated MMAs (Figure 5B). Conversely, the model-predicted MMAs of the caudal origin were generally smaller, but the pattern of kinematic peaks and troughs did usually correspond with the experimental pattern (Figures 5C,G).

At the glenohumeral joint (Figure 6), the cranial-most origin showed some consistency in sign: both SIMM model and experimental data estimated negative MMAs in abduction-adduction (i.e., adductor), generally negative MMAs in flexion-extension (i.e., extensor), and generally positive MMAs in long axis rotation (i.e., pronator). However, the experimental estimates for flexion-extension and long-axis rotation occasionally crossed zero (Figure 10I; i.e., some small moment arms for glenohumeral flexion and external rotation/supination). Magnitudes of the MMAs overlapped somewhat in abduction-adduction and long-axis rotation, and showed generally similar patterns of peaks and troughs (Figure 10C), but flexion-extension MMAs were more obviously dissimilar in both magnitude and general pattern. Rank orders of MMAs also did not agree: the largest peaks of experimentally-estimated MMAs were in adduction, then internal rotation, then extension, whilst the model-predicted MMAs were approximately similar (Figure 10I).

The glenohumeral joint MMAs of the caudal-most origin (Figure 6, Figures 10D,J) were consistent in sign, magnitude and rank order; i.e., this part of the muscle is interpreted in both model-predicted and experimental estimates as primarily a humeral internal rotator, with smaller MMAs for humeral adduction and extension.

#### M. Latissimus Dorsi

The m. latissimus dorsi partially originates from the scapula, and partially from a broad attachment along the thoracic vertebrae. The scapular head of m. latissimus dorsi only crosses the glenohumeral joint, whilst the remainder also crosses the scapulocoracoid-clavicle-interclavicle joint.

For the scapular head of m. latissimus dorsi at the glenohumeral joint, the experimentally-estimated MMAs and model-predicted MMAs agreed well (Figure 7). The MMAs were consistent in sign, though there was more negative (i.e., extension) MMAs seen in experimental data than in the model (Figure 7, and Figures 10E,K). The MMAs also agreed in approximate magnitude and rank order. Thus, the interpreted actions and absolute and relative leverages of m. latissimus dorsi (scapular head) are consistent between experimental and SIMM model methods; i.e., primarily a humeral internal rotator, but with large moment arms for humeral abduction and flexion.

The portion of m. latissimus dorsi originating from the thoracic vertebrae was implanted at the level of T6, and compared to the modelled muscle line of action also originating from this vertebra. At the scapulocoracoid-clavicle-interclavicle joint, experimentally-estimated and model-predicted MMAs were consistent in sign (acting to rotate the scapulocoracoid medially) and had similarly large magnitudes (Figures 5D,H). However, the pattern of kinematic peaks and troughs were not overtly consistent in every trial (Figure 5D).

For the vertebral origin of m. latissimus at the glenohumeral joint, MMAs agreed in sign for flexion-extension (positive; glenohumeral flexion) and long-axis rotation (positive; internal rotation). However, the smaller MMAs in abduction-adduction fluctuated either side of zero, with positive (abduction) experimental estimates but generally negative (adduction) SIMM model predictions (Figure 7, and Figures 10F,L). The magnitudes of flexion-extension and long-axis rotation MMAs were similar (Figure 7 and Figure 10L), and the pattern of kinematic peaks and troughs (or lack thereof, for abduction-adduction) also agreed somewhat (Figure 10F). The rank order of peak MMAs was consistent, with the interpreted actions of m. latissimus (mid-vertebral portion) for both SIMM model and experimental data being primarily internal humeral rotation and glenohumeral flexion.

### Muscle Architecture and Torque

**FIGURE 12:**
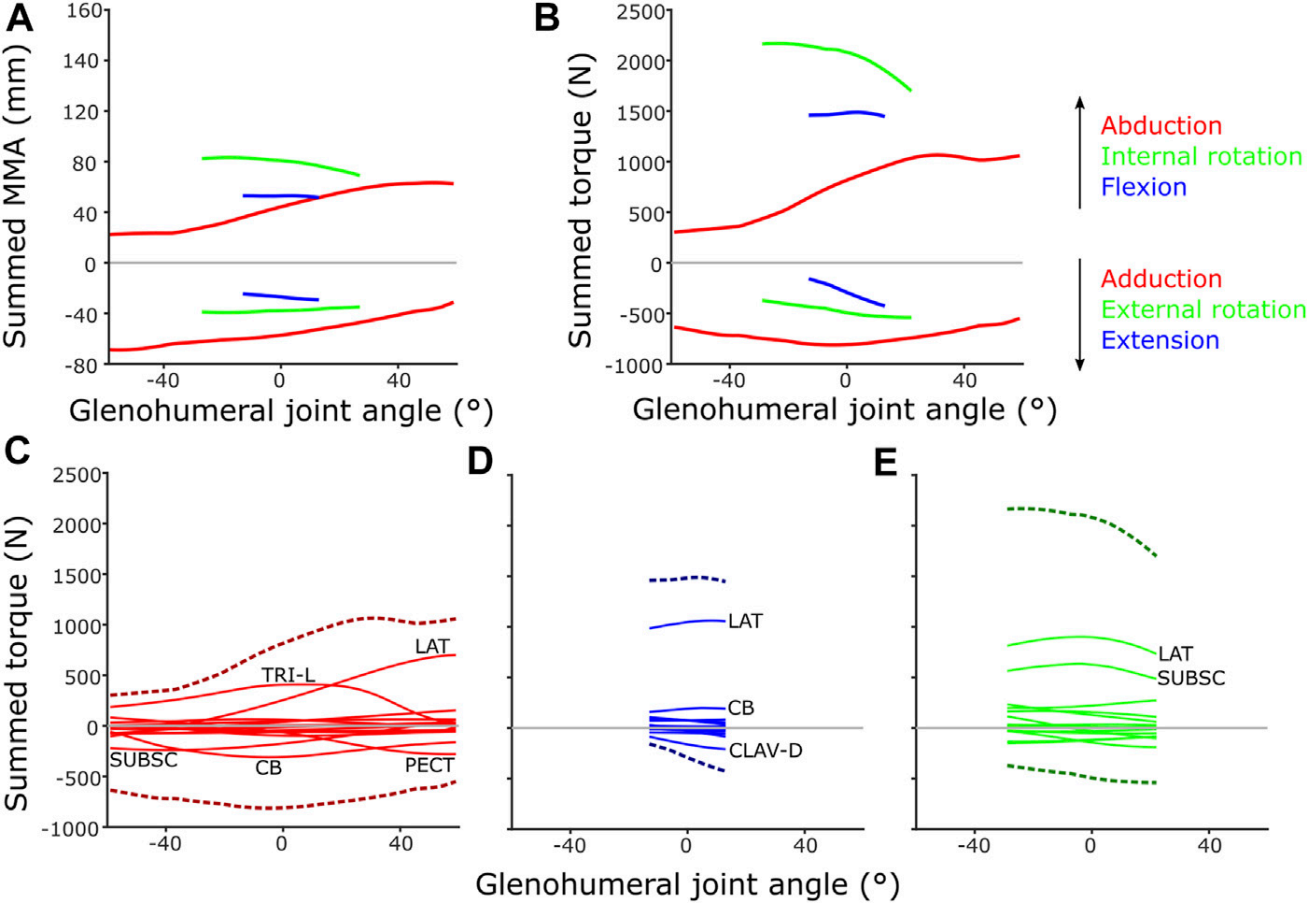
Model-predicted MMAs and muscle torques at the glenohumeral joint, using the updated SIMM model muscle pathways from diceCT and for single-axis DOF rotations: **(A)** Summed MMAs, **(B)** Summed torques. Major muscles contributing to summed torque (dashed lines) are labelled for each rotational DOF: **(C)** Abduction-adduction, **(D)** Flexion-extension, **(E)** Internal-external rotation. LAT = m. latissimus dorsi, TRI-L = m. triceps brachii longus, SUBSC = m. subscapularis, CB = m. coracobrachialis, CLAV-D = m. clavodeltoideus. For details on individual muscles, see the Supplementary Material.

Inclusion of muscle architectural parameters in the updated SIMM model to estimate muscle torque at different joint angles generally yielded similar patterns to MMAs on the individual muscle level (Supplementary Figures S1–S3 vs. Supplementary Figures S6–S8). Occasionally, the differing physiological cross-sectional area (PCSA) of muscle parts with otherwise similar MMAs yielded differing, higher torques (e.g., m. coracobrachialis longus compared to m. coracobrachialis brevis, Supplementary Figure S2 vs. Supplementary Figure S7), as would be expected. Patterns between summed muscle MMAs and torques were also generally quite similar across the glenohumeral joint (Figures 12A,B). For instance, inclusion of architecture and calculation of muscle torque highlighted the predominance of some joint movements, compared with MMAs alone, e.g., the relative magnitude of internal humeral rotation, which is ranked largest in both summed MMAs and torques at the glenohumeral joint.

However, some patterns differed between summed MMAs and torques: across the abduction-adduction ROM, torque values peak around the middle of the glenohumeral abduction-adduction range (i.e., a neutrally-positioned glenohumeral joint), compared with MMAs which peak at extremes of abduction and adduction. This is likely due to the modelled fibre lengths exceeding the optimal length for force production at extreme joint angles. The rank order of peak summed MMAs and muscle torques at the glenohumeral joint also differ in some respects, too. Rank order was maintained in terms of peak MMA and torque, except adduction and flexion which swap position as second and fourth-ranked. MMAs suggested humeral adduction to be fairly important (with peak MMAs ranked second, below internal humeral rotation) due to the large-summed adduction MMAs in the -60° abducted joint (Figure 12A), but the summed peak adduction torque is relatively lower (ranked fourth) (Figure 12B).

If the torques of individual muscles are plotted against summed torque, the contributions of each muscle can be evaluated (Figures 12C–E). These show that MMA alone is occasionally not the best predictor of a muscle’s contribution to limb function. For example, m. biceps brachii has large humeral adductor moment arms (Supplementary Figure S2) and might be anticipated to be a major contributor to adduction, but in terms of torque it is overshadowed by m. pectoralis, m. coracobrachialis and m. subscapularis due to their larger PCSAs (Figure 12C).

## Discussion

Here, we critically evaluate the behaviour and outputs of an echidna forelimb musculoskeletal model in terms of its predicted ranges of motion, muscle path accuracy, and muscle moment arms. The echidna’s unusual body plan and resultant biomechanical/locomotory function provides an opportunity to validate established model-building practices beyond those applied to more “conventional” anatomical configurations [i.e., erect bipeds such as humans and avian/non-avian dinosaurs (e.g., Bishop et al., 2021a), sagittal/erect quadrupeds such as therian mammals (e.g., Stark et al., 2021)]. Our initial model was constructed using a traditional workflow, often used to model extinct animals, with our primary data consisting of bone morphology and muscle attachment points from the literature (Regnault and Pierce, 2018). In the present study, we aimed to 1) validate the modelling process and its outputs in a species with “unconventional” anatomy and posture, and 2) identify lessons that could be learned and applied to future models interpreting function in extant and extinct quadrupedal animals. We also explored whether the addition of a further level of anatomical detail (muscle architecture; generally unavailable for extinct animals) alters the functional interpretations made from muscle moment arms alone.

### A Light-Touch Approach can Generate Reasonably Accurate Muscle Paths

Our study identifies several key lessons that can be applied to future modelling studies. Firstly, a light-touch approach to muscle modelling – using only muscle attachment sites plus the fewest “wrap objects” necessary to avoid muscle-bone interactions through single-axis DOF rotations – can generate reasonably accurate muscle paths. In comparing the muscle pathways of the initial model (Regnault and Pierce, 2018) to the pose-matched digitally dissected specimen, we found representative muscle paths were generally well-reproduced. Where we found muscle paths to display inaccuracy compared with the 3D reconstructed soft tissues, correction of those inaccuracies shows the effects on predicted muscle moment arms to be generally minimal (see Supplementary Figures S1–S3).

The initial model’s muscle attachment coordinates were estimated from images and descriptions of Gambaryan et al. (2015). Unsurprisingly these coordinates occasionally fell outside of our specimen’s specific attachment areas (identified via diceCT; Regnault et al., 2020), usually due to small or narrow attachment sites, e.g., m. latissimus dorsi scapular origin. The adjustments made to either muscle attachment sites or wrap objects are summarised in Supplementary Table S1, and their impact on estimated MMAs (around a single rotational DOF) are shown in Supplementary Figures S1–S3. For most muscles, the effects of adjusting muscle paths were negligible: small changes in moment arm magnitude, or the pattern of moment arm change with joint angle. The signs of MMAs (i.e., positive or negative) and rank order of MMAs (i.e., whether abduction-adduction, flexion-extension, or long-axis rotation are largest or smallest) were unchanged for most muscles.

There were a few exceptions. M. pectoralis was originally modelled originating from the interclavicle to the third sternebra and interpreted to have regional variation in its action cranially vs. caudally (Supplementary Figure S2; see also Regnault and Pierce, 2018). Adjustment of its origin to a narrower site caudally, from the manubrium to the third sternebra, now shows it acting more homogenously (i.e., still a humeral pronator and extensor, but perhaps also intrinsic stabilisation of adduction-abduction). M. biceps brachii longus appeared to originate principally from the coracoid, rather than epicoracoid as originally described by Gambaryan et al. (2015); as a result, the small lateral-rotation moment arm contributed by this muscle at the scapulocoracoid-clavicle-interclavicle joint is no longer present in the revised model (Supplementary Figure S1). It is not clear whether this represents intraspecific variability; further sampling would be beneficial.

There were some adjustments to muscle paths (via their wrap objects) which could also change the inferences of functional aspects. At the glenohumeral joint, this altered the sign of some MMAs at certain joint angles and therefore aspects of the inferred muscle action: mm. biceps brachii brevis and longus changed from having minor action as humeral external rotators (supinators) to mostly internal rotation (pronation), and m. biceps brevis additionally from humeral extensor to flexor (Supplementary Figure S2); m. acromiodeltoideus changed from having flexor-extensor actions to just extensor; and m. clavodeltoideus changed from having action as an adductor to both abduction-adduction (Supplementary Figure S2). However, the affected moment arms were all relatively small (i.e., closer to zero) in both initial and adjusted versions of the model, as compared with the much larger humeral adductor moment arms (interpreted as the principal action of m. biceps brachii), extensor/external rotator moment arms (the principal action of m. clavodeltoideus) and extensor/abductor moment arms (the principal action of m. acromiodeltoideus).

For the majority of muscles, differences in paths generated by the initial “light-touch” model and real specimen’s digitally-dissected muscle meshes were small, and any adjustments made had little impact on their leverage, interpreted action(s), and relative importance of each action. For future models – particularly fossils, where 3D muscle geometry is unknown – this means researchers can have reasonable confidence in a minimalist approach that uses osteological correlates of attachment and as few modifications (wrap objects) as necessary to generate realistic muscle paths.

### Joint Translations and Multi-Axis Rotations Maximise Joint Range of Motion

A second key lesson of this study is that use of independent, single-axis DOF rotations to determine the limits of joint range of motion (ROM) is unlikely to capture the full picture, at least at some joints. In our initial model, the limits to joint ROM were first predicted by rotating around a single joint axis (e.g., flexion-extension) until bone-on-bone contact (Regnault and Pierce, 2018). This method of assessing ROM – single rotational DOF, with no joint translation – is customary within fossil modelling studies (see Bishop et al., 2021c), and used to exclude impossible poses when reconstructing extinct animals. Our experimental data show that, converse to expectations, some of the movements possible in a real, intact animal can exceed the model’s osteological “limits” when predicted this way. Given that osteological limits predicted by such models are generally accepted to represent the maximum mobility possible – ligaments, muscles, and skin should all act to constrain mobility in the real specimen (Hutson and Hutson, 2012; Arnold et al., 2014) – our result was unexpected, however not without precedent (Hutson and Hutson, 2014, 2015).

More recently, automated and iterative workflows have been developed that evaluate ROM through multiple DOF simultaneously (Manafzadeh and Padian, 2018; Richards et al., 2021). Manual checking must be performed to ensure biologically implausible poses are not included (Regnault and Pierce, 2018; Bishop et al., 2021c), but these methods have the potential to more realistically represent mobility (since animal movement rarely occurs through pure rotation about a single axis), and allow for interactions between DOF that could expand or limit ROM in an informative manner to researchers. These workflows (Manafzadeh and Padian, 2018; Richards et al., 2021), and several other studies (Pierce et al., 2012; Lai et al., 2018), include joint translation, but in general translations are usually not included in models (Bishop et al., 2021a; Wiseman et al., 2021) and there are even fewer experimental data examining the effects of joint translation on ROM (e.g., Baier and Gatesy, 2013; Tsai et al., 2020; Manafzadeh and Gatesy, 2021), even though they are vital to validate such models.

Our study suggests that joint translation is an important component of joint mobility, alongside simultaneous rotational DOF. Mobility measured experimentally exceeded the model’s ROM predictions for glenohumeral flexion-extension and internal-external rotation, and antebrachial abduction-adduction and internal rotation, whilst other types of mobility were below model-predicted ROMs (glenohumeral abduction-adduction, humeroradioulnar flexion-extension). Other studies have also found that exclusion of joint translation from models can both under- and over-estimate true ROM (Hutson and Hutson, 2014, 2015; Manafzadeh and Gatesy 2021), though these studies examine only archosaur species and primarily find over-estimation only to be the case for specific joint morphologies (bi-condylar or gliding/planar: ostrich wrist, alligator knee and ankle, guineafowl knee and ankle).

The effect of translation on experimental ROM in our study can be inferred on the 3D plots by the width of envelope at the point where other rotational joint angles are zero. For example, at the hemi-sellar glenohumeral joint, it can be seen that the flexion-extension envelope is wider than the model predicted, even when long-axis rotation and abduction-adduction are zero (Figure 4). The almost tripling of flexion-extension ROM (from 26° in the initial model, to up to a possible maximum of 72° across all experimental trials; Table 1) can be explained by translation at the joint surface. Cranio-caudal translations at the glenohumeral joint (which would act to increase flexion-extension) were large: recorded up to a maximum of 10.4 mm (mean = 7.5 mm) and distributed relatively evenly between cranio-caudal movements, due to sliding along the echidna’s elongate hemi-sellar glenoid morphology. For context, the cranio-caudal length of the glenoid in the modelled echidna is approximately 12.2 mm.

The effect of simultaneous rotations on experimental ROM can also be seen on the 3D plots (though the contributory effect of rotations cannot be separated from translations in these experimental data, and so other rotational axes could also be contributing here). For example, although the experimental data suggest glenohumeral abduction-adduction ranges close to the model’s predicted limits, greatest humeral adduction could only be achieved concomitant with humeral extension (Figure 4); likewise, greatest humeral abduction appears to necessitate some flexion. An interesting secondary point is that the experimental data do support the very large glenohumeral joint abduction-adduction range predicted by the initial model. Therefore, even in cases where ROM is presumed to be highly constrained based on articular morphology, soft tissues, and available *in vivo* data, such as in the echidna (Jenkins, 1970; Luo, 2015; Regnault and Pierce, 2018), models and validation studies such as ours can be valuable in challenging our assumptions about what is (or is not) possible.

At the modified-condylar humeroradioulnar joint, the dramatic amount of antebrachial internal rotation seen experimentally as compared to the initial model (55% of the total long-axis rotation ROM in the initial model vs 96% experimentally) seems to be due, in part, to translation (Figure 4; Table 1) along the single, elongate ball-like humeral condyle. However, to achieve maximum internal rotation, adduction of the antebrachium is also required (Figure 4). The increase in abduction-adduction measured experimentally compared to the initial model’s ROM “limit” appears similarly due to the interactions of multiple DOF: for instance, maximum abduction is only achieved experimentally alongside internal rotation and extension (Figure 4), and may be assisted by pre-postaxial translations up to a possible maximum of 7.6 mm along the ulnar articular surface (mean = 4.5 mm), again distributed relatively evenly between pre- and postaxial movements.

More expectedly, the experimentally-estimated ROM at the scapulocoracoid-clavicle-interclavicle joint was less than the initial SIMM model-predicted ROM (Figure 3A). This joint is comprised of two articulations in the echidna (between the acromion of the scapulocoracoid and the fused clavicle-interclavicle laterally, and between the coracoid and interclavicle ventrally), shown in Figure 3A. The effect is to essentially produce a single rotational axis, i.e., only a single DOF, unlike the glenohumeral and humeroradioulnar joints, which had opportunities for interactions between several DOF (including rotations and translations).

The experimental ROM at the scapulocoracoid-clavicle-interclavicle joint occupies a more medial range of rotations than the SIMM model “limit” (Figure 3A, black arc), suggesting additional factors are relevant in the intact animal compared with a digital model. For instance, loading of the forelimb in the intact echidna is likely to result in a dorsally- and medially-directed force at the glenoid (due to the lateral and ventral orientation of the humerus/glenoid; Pridmore, 1985), pulling the acromion-clavicle and coracoid-interclavicle joint surfaces apart. Alterations to model joint spacing can alter joint ROM estimates (e.g., Brassey et al., 2017), and increased spacing has been shown in the echidna model to increase estimated ROM (albeit at the glenohumeral joint; Regnault and Pierce, 2018).

Thus, future models may need to explore not only static joint spacing choices in the re-articulation of bones, but also consider how joint spacing may differ in a loaded limb (e.g., through sensitivity analyses). Interestingly, the recent study of Manafzadeh and Gatesy (2021) shows inclusion of a single translational DOF in distraction-compression (equivalent to increasing or decreasing joint spacing) results in great improvement of model-predicted osteological limits, to encompass most possible *ex vivo* and *in vivo* joint poses. Taken together, our results suggest translation to be a potentially important component to ROM estimates in addition to simultaneous rotational DOFs. However, the decision to include further translational DOF (beyond distraction-compression AKA appropriate joint spacing) is one to be made case-by-case on a joint-morphology and species-specific basis, guided by data from extant animals.

### Independently-Calculated Experimental MMAs Validate Model Predictions

We also found that under an identical kinematic regime, experimentally-derived MMAs calculated using a geometric method reasonably matched the model-predicted MMAs estimated using partial velocity. Of the six muscles evaluated (and eight muscle paths total), moment arms for five muscles/paths agreed well in sign, general kinematic pattern, magnitude and rank order, thus validating predicted muscle function: m. clavodeltoideus (Figures 5A,E, 8, 10A,G), m. triceps longus superficialis (Figures 9, 11B,D,F,H, Supplementary Figure S5), m. biceps brevis (Figures 9, 11A,C,E,G, Supplementary Figure S5), m. latissimus dorsi (scapular origin) (Figures 7, 10E, K), and m. pectoralis (caudal origin) (Figure 5C,G and Figures 6, 10D,J).

The MMAs for the remaining three muscle paths agreed less well crossing at the glenohumeral joint: m. latissimus dorsi (vertebral origin) (Figure 10F), m. coracobrachialis longus (Figures 8, 10B), and m. pectoralis (cranial origin) (Figure 10C). However, despite discrepancies between experimentally-derived and model-predicted MMAs for some muscles, our data showed aspects of the model outputs that are still informative for the purposes of functional interpretation. The sign (positive or negative), approximate magnitude, and/or rank order of MMAs agreed for some of these muscles, even when the MMA kinematic patterns did not. These MMA parameters provide a useful guide for inferring muscle action(s) – for example, whether a muscle (such as m. latissimus dorsi, vertebral origin; Figure 10F) is interpreted as a glenohumeral flexor vs. extensor (MMA sign), its approximate leverage (MMA magnitude), and whether it is considered primarily an internal rotator vs. flexor vs. abductor (MMA rank order).

There is clear utility in understanding why some muscles agreed well between methods and others did not in order to build confidence in our interpretations of future models. Unfortunately, it is not clear from our data which factors are most relevant. Since the experimental method of calculating MMAs uses a straight line of action between implanted muscle markers, disagreement could presumably be related to muscles undergoing more complex trajectories within the model. However, MMA agreement does not appear to be related to the number of joints crossed by a muscle; uni- and bi-articular muscle paths did not clearly differ in degree of agreement. MMA agreement is also not clearly related to the complexity of muscle wrapping objects; although some muscles without wrap objects had better agreement (m. clavodeltoideus, m. triceps, m. latissimus scapular head, m. pectoralis caudal origin) others had worse (m. pectoralis cranial origin), and yet others with several wrap objects agreed fairly well (m. biceps brevis). Another plausible factor could be whether muscles change direction near to their attachment site or the joint of interest, but again examples for and against this are seen in the muscles evaluated here, and so a clear relationship cannot be established. A final possibility could be error related to marker placement: approximate marker locations were checked on micro-CT scans post-data collection (though the muscles could not themselves be visualised), and on subsequent dissection markers were noted if found to be displaced (as for the muscles discounted in this study due to obvious marker migration). However, dynamic marker migration during data collection may be possible and a potential source of error, despite steps taken to secure them (use of smallest gauge needle possible, tissue glue).

However, the very close agreement of many muscles between the experimentally-derived MMAs and the SIMM model-predicted MMAs provides important alternate validation for different conceptual methods of calculating MMAs, and the comparison of MMAs calculated using different methodologies. There are several ways to define and calculate MMAs, both by models (see Sherman et al., 2013) and experimentally (An et al., 1984). The SIMM model uses a partial velocity method (Delp and Loan, 1995), whilst the script we developed for processing experimental data uses a geometric method (based on the perpendicular distance between joint centre and muscle line of action). A substantial body of research suggests that model-predicted MMAs fall across similar ranges to experimental estimates (typically made via the “tendon travel” method) (see Brassey et al., 2017). Here, we find important confirmation that this is also the case for experimental estimates made via geometric calculation (particularly for muscles with straight lines of action), and apparently avoiding some potential pitfalls of experimental tendon travel estimates, such as kinematic cross-talk (Hutchinson et al., 2015). While there is room for refinement of the geometric method – most saliently, in accounting for less straightforward muscle paths – the broad agreement between multiple techniques can give researchers further confidence in 3D musculoskeletal models of animals with diverse morphologies.

### Muscle Architecture can Alter Functional Inferences

Finally, addition of muscle architecture data alongside muscle moment arms has capacity to change aspects of the functional inferences made from a model at a finer scale (Bates and Falkingham, 2018). In a previous study, the short-beaked echidna was found to exhibit little variation in normalised architectural parameters of its forelimb muscles (Regnault et al., 2020). In this study, we therefore anticipated that inclusion of architectural data (to yield muscle torques) would not greatly affect our conclusions, compared to using MMAs alone. This was often the case – particularly at the individual muscle level, muscle torque vs. joint angle exhibited generally similar patterns to MMA vs joint angle (Supplementary Figures S1–S3 vs. Supplementary Figures S6–S8).

However, when evaluating summed muscle torque or individual muscle contributions to summed muscle torque, inferences can differ compared with those made from MMAs alone. From the initial model (Regnault and Pierce, 2018), m. biceps brachii was inferred to be particularly important in supporting the echidna’s sprawling posture and locomotion, due to the large humeral adduction moment arms of this muscle. However, when architecture data are added as a model parameter, the inferred role of m. biceps brachii is diminished compared to other muscles (m. pectoralis, m. subscapularis) due to their large muscle volumes and resultantly large physiological cross-sectional areas. Further, while our summed muscle torques support the inference that the echidna’s forelimb musculoskeletal anatomy is optimised for humeral internal rotation (Figure 12B), summed muscle torques in flexion outrank those for adduction, a pattern that differs from results based solely on MMAs (Regnault and Pierce, 2018). Aside from adduction and flexion, the rank orders of peak values for other movements (internal rotation, abduction, external rotation and extension) are the same between summed MMAs and muscle torques. The change in relative importance of adduction and flexion reflects the cumulative effect of smaller differences in individual muscles, particularly the combination of the large PCSA and large MMAs of m. latissimus in glenohumeral flexion (Figure 12D, Supplementary Figures S2, S7; see also Regnault et al., 2020).

In extinct animals, soft tissues, such as muscles, are not preserved in sufficient detail to allow direct measurement of these functionally-relevant parameters. In absence of detailed architectural parameters, less detailed parameters such as muscle volume (size) can still allow for some refinement of inferences based on MMA alone, such as for m. biceps brachii, discussed above. Diverse data from living animals can help to further guide estimates of architectural parameters (Bates and Falkingham, 2018; Bishop et al., 2021a) or even direct evidence-based reconstructions of these parameters for extinct species (Fahn-Lai et al., 2020). Here we have used generalised curves for muscle and tendon properties, and estimations of torque over the full ROM can be sensitive to these properties. Further work may be needed to evaluate the relationship between MMA and torque in modelling studies, ideally with species-specific values measured on fresh muscle tissue under controlled conditions. However, our findings in this study affirm the need to account for muscle architecture to some degree in musculoskeletal computer models (if possible) or to recognise architecture as a source of disparity in functional model outputs (where not possible).

## Conclusions

Our study has several pertinent findings for the field of musculoskeletal computer modelling. Firstly, we find that a minimalist muscle-wrapping approach – in other words, one that minimises assumption or knowledge of muscle anatomy beyond attachment site – can approximate muscle geometry. Additionally, when further intervention is required, and muscle attachment or geometry is adjusted (as was occasionally the case in our echidna model), the resultant effect on MMAs appears minimal. This approach could be of particular utility in situations where muscle anatomy may not be clearly characterised, for example, in extinct or difficult-to-source extant animals.

We find joint ROMs from experimentally-manipulated cadavers to have similar ranges to those predicted using single-axis DOF osteological ROM in most directions, including the surprisingly wide range of humeral abduction-adduction initially predicted by the model. However, there are also several discrepancies, the biggest being ROM in flexion-extension at the glenohumeral joint and long-axis rotation at the humeroradioulnar joint, where the experimental ROM far exceeded the model-predicted ROM. These results further support the contention that simultaneous rotational and translational DOF can expand the envelope of possible joint poses in certain anatomical directions, and should be accounted for in model design, if possible. Importantly, the experimental data collected here provides important insights into joint function that can be used in the future to refine osteological ROM modelling assumptions and methodological development.

We also find high-level agreement for most muscles between experimentally-derived and SIMM model-predicted MMAs. Our geometric method of estimating experimental MMAs from implanted muscles appears equivalent to model-predictions made via the partial velocity method, particularly for muscles with straightforward paths. The geometric method and tools we have developed here may have further utility and application where other methods (such as tendon travel) fall short: for instance, proximal limb muscles, those with little tendon, and joints with 3D mobility (where kinematic cross-talk may be a concern) (e.g., Hutchinson et al., 2015; Brassey et al., 2017). The method allows for the creation of simplified musculoskeletal models in Maya or other software to calculate 3D MMAs, where previously such models may have explored only planar MMA calculation (e.g., Regnault et al., 2017).

Finally, we observe that inclusion of muscle architecture within models can change some functional interpretations of muscle roles, and MMAs alone may not yield a complete functional signal, echoing the caveats of other studies. However, for the muscles we model here, patterns of MMAs and muscle torques across joint angles are similar at an individual level. Their contributions to summed torques vs. summed MMA can differ, particularly for muscles with large PCSA, which could impact the rank order of peak summed values. Nonetheless, our addition of muscle architecture supports a major conclusion drawn from the initial study based on MMAs alone: that the forelimb musculoskeletal system of the echidna is specialised for humeral internal rotation, consistent with *in vivo* locomotion data.

## Data Availability

The raw data supporting the conclusions of this article will be made available by the authors, without undue reservation.
