# Supplementary material for: Validation of an Echidna Forelimb Musculoskeletal Model Using XROMM and diceCT
Source: Front Bioeng Biotechnol. 2021 Nov 8;9:751518. doi: 10.3389/fbioe.2021.751518 (PMC8606742; doi:10.3389/fbioe.2021.751518)

**Supplementary Material**

**Title:** Validation of an echidna forelimb musculoskeletal model using XROMM and diceCT

**Authors:** Sophie Regnault^1,2,*^, Philip Fahn-Lai^1,3^, Stephanie E. Pierce^1,*^

^1^Museum of Comparative Zoology and Department of Organismic and Evolutionary Biology, Harvard University, Cambridge, MA 01239, USA

^2^Institute of Biological, Environment & Rural Sciences, Aberystwyth University, Aberystwyth, Ceredigion, SY23 3DA, UK

^3^Concord Field Station and Department of Organismic and Evolutionary Biology, Harvard University, Bedford, MA 01730, USA

*Corresponding authors: Sophie Regnault (sor24@aber.ac.uk) and Stephanie E. Pierce (spierce@oeb.harvard.edu)

**Supplementary Table S1**: Adjustments to SIMM model muscle attachments and wrap objects following DICECT digital segmentation of the echidna specimen’s forelimb musculature. The SIMM model muscle attachment coordinates are given as millimetres relative to the segment’s joint centre. For example, to interpret adjustments to the origin of m. latissimus dorsalis part 4 (scapular head): the origin coordinates in the initial model were (-29.015, -2.536, 37.245) relative to the scapular segment, and these were adjusted to (-33.204, -3.807, 36.117), representing a move of approximately -4mm, -1.3mm and -1mm along the scapular x,y and z axes, respectively. Muscles whose anatomical attachments differed appreciably from the observations of Gambaryan et al (2015) and our initial model are denoted by an asterisk*, and discussed in-text.

| **Muscle** | **Attachment site adjustment** | **Wrap object adjustment** |
| --- | --- | --- |
| m. latissimus dorsi, scapular head (“part 4”)  (Regnault et al., 2020 Fig 3) | Scapular segment origin from  (-29.015, -2.536, 37.245)  to  (-33.204, -3.807, 36.117) |  |
| m. pectoralis (“parts 1-3”)*  (Regnault et al., 2020 Fig 4) | Body segment origins (parts 1-2) respectively from  (12.068, 0.103, -2.827)  (-3.944, 2.377, -12.004)  to  (-3.944, 2.377, -12.004)  (-9.264, 5.73, -19.426)  and  Humeral insertion (parts 1-3) all from  (7.639, -1.76, -16.531)  to  (5.512, -2.25, -14.352) |  |
| m. clavodeltoideus  (Regnault et al., 2020 Fig 3 and 4) | Clavicle-interclavicle origin from  (19.706, 11.6, 0.688)  To  (22.342, 11.972, 2.877)  And  Humeral insertion from  (8.881, 0.659, -10.035)  To  (6.628, 10.842, -14.24) |  |
| m. spinodeltoideus  (Regnault et al., 2020 Fig 4) | Humeral insertion from  (6.527, 4.729, -10.313)  To  (6.184, 10.428, -12.855) | Ellips_proxhum_delts  (Ellipsoid wrapping object, translation and rotation coordinates and radius adjusted) |
| m. acromiodeltoideus  (Regnault et al., 2020 Fig 3 and 4) | Clavicle-interclavicle origin from  (0.642, 5.229, 0.699)  To  (1.225, 3.697, -0.635)  And  Humeral insertion from  (5.481, 4.116, -7.6)  to  (4.314, 9.247, -8.799) | Ellips_proxhum_delts  (Ellipsoid wrapping object, translation and rotation coordinates and radius adjusted) |
| m. infraspinatus (“parts 1-3”)  (Regnault et al., 2020 Fig 4) | Humeral insertion (parts 1-3) from  (8.588, -3.563, -12.389)  to  (7.947, 0.667, -6.495) | Ellips_proxhum_delts  (Ellipsoid wrapping object, translation and rotation coordinates and radius adjusted) |
| m. supraspinatus  (Regnault et al., 2020 Fig 4) | Humeral insertion from  (7.702, -5.658, -9.012)  to  (6.591, -5.27, -6.51) | Ellips_proxhum_infra  (Ellipsoid wrapping object, translation and rotation coordinates and radius adjusted;  this wrap object was made inactive for m. infraspinatus, for which it no longer interacted with). |
| m. teres minor  (Regnault et al., 2020 Fig 3 and 4) | Scapular segment origin from  (-8.791, -0.551, -0.045)  to  (-13.909, 2.966, -2.305)  and  Humeral insertion from  (-12.588, 3.02, -1.61)  to  (-15.548, 3.695, -0.924) | Ellips_scap_teresmin  (This wrap object was deleted, as m. teres minor no longer interacted with it) |
| m. teres major  (Regnault et al., 2020 Fig 3 and 4) | Scapular segment origin from  (-31.579, -1.783, 37.423)  to  (-30.982, -1.912, 36.048)  and  Humeral insertion from  (-16.608, 12.801, -5.204)  to  (-19.182, 12.118, -6.352) |  |
| m. scapscapularis (“parts 1-3”)  (Regnault et al., 2020 Fig 4) | Humeral insertion (parts 1-3) from  (-16.363, -1.333, -1.419)  to  (-15.841, -0.39, 0.209) |  |
| m. coracobrachialis brevis  (Regnault et al., 2020 Fig 4) | Scapular origin from  (-24.736, 15.056, -18.769)  to  (-25.47, -12.633, -16.298) |  |
| m. coracobrachialis longus  (Regnault et al., 2020 Fig 3 and 4) | Scapular segment origin from  (-26.782, -16.485, -17.636)  to  (-27.595, -14.3, -17.218)  and  Humeral insertion from  (-10.201, 29.432, -15.056)  to  (-15.0572, 32.1839, -16.0182) | Tor_hum_coracolong  (Torus wrap object, translation and rotation coordinates and radius adjusted) |
| m. biceps brevis  (Regnault et al., 2020 Fig 3 and 5) | Scapular segment origin from  (-15.819, -19.783, -12.869)  to  (-27.064, -14.763, -17.876)  and  Lower limb (radial) insertion from  (18.354, -7.704, -18.068)  to  (14.348, -3.863, -14.332) | New wrap object created  Tor_hum_biceps_2  (Torus wrap object created for this muscle path) |
| m. biceps longus*  (Regnault et al., 2020 Fig 3) | Epicoracoid segment origin from  (-1.01, -8.424, 0.687)  to scapular segment origin  (-20.322, -14.913, -18.072)  and  Lower limb insertion from  (17.317, -0.806, -13.452)  To  (17.317, -0.806, -13.452) | Tor_hum_biceps  (Torus wrap object, translation and rotation adjusted) |
| m. subcoracoideus | Epicoracoid segment origin from  (6.3602, -8.2360, 2.7451)  to  (3.395, -2.158, 2.204) | Ellips_epi_subcora  (Ellipsoid wrapping object, translation and rotation coordinates and radius adjusted)  Ellips_epi_subcora2  (Ellipsoid wrapping object, translation and rotation coordinates and radius adjusted) |

**Supplementary Table S2:** Root Mean Square Error (RSME) between the SIMM model and experimental MMA data were calculated as described in the Materials and Methods. RSME was then normalized in four different ways by dividing it by the mean (Mean nRMSE), the range (Range nRMSE), the standard deviation (s.d. nRMSE), and the interquartile range of values (i.q.r. nRMSE).

| Muscle | Joint | DOF | RMSE | Mean nRMSE | Range nRMSE | s.d. nRMSE | i.q.r. nRMSE |
| --- | --- | --- | --- | --- | --- | --- | --- |
| m. biceps brachii brevis | Glenohumeral | Abduction-adduction | 4.16 | -0.32 | 0.42 | 2.07 | -1.92 |
|  |  | Long axis rotation | 2.83 | 1.80 | 0.21 | 0.63 | -0.42 |
|  |  | Flexion-extension | 2.01 | 0.50 | 0.34 | 1.89 | -1.34 |
|  | Humeroradioulnar | Abduction-adduction | 3.10 | -0.67 | 0.28 | 0.88 | -0.56 |
|  |  | Long axis rotation | 6.67 | -46.87 | 0.31 | 1.43 | -0.92 |
|  |  | Flexion-extension | 4.20 | -0.31 | 0.30 | 1.00 | -0.66 |
| m. coracobrachialis longus | Glenohumeral | Abduction-adduction | 1.59 | -0.13 | 0.14 | 0.43 | -0.34 |
|  |  | Long axis rotation | 7.90 | -9.49 | 0.39 | 1.33 | -1.04 |
|  |  | Flexion-extension | 7.92 | 0.72 | 0.57 | 3.90 | -3.03 |
| m. clavodeltoideus | Glenohumeral | Abduction-adduction | 0.56 | 0.22 | 0.08 | 0.17 | -0.10 |
|  |  | Long axis rotation | 1.77 | -0.27 | 0.24 | 0.57 | -0.38 |
|  |  | Flexion-extension | 1.48 | -0.17 | 0.17 | 0.44 | -0.36 |
|  | Scapulocoracoid-clavicle-interclavicle | Medial-lateral rotation | 0.60 | -0.06 | 0.12 | 0.25 | -0.15 |
| m. latissimus dorsi (vertebral origin) | Glenohumeral | Abduction-adduction | 11.83 | 2.45 | 0.44 | 4.33 | -6.05 |
|  |  | Long axis rotation | 9.07 | 0.30 | 0.20 | 0.50 | -0.52 |
|  |  | Flexion-extension | 8.84 | 0.45 | 0.19 | 0.46 | -0.28 |
|  | Scapulocoracoid-clavicle-interclavicle | Medial-lateral rotation | 9.80 | 0.22 | 0.27 | 0.83 | -0.69 |
| m. latissimus dorsi (scapular origin) | Glenohumeral | Abduction-adduction | 2.82 | 0.21 | 0.13 | 0.37 | -0.24 |
|  |  | Long axis rotation | 5.92 | 0.20 | 0.44 | 1.90 | -1.32 |
|  |  | Flexion-extension | 5.80 | 1.64 | 0.12 | 0.30 | -0.21 |
|  | Scapulocoracoid-clavicle-interclavicle | Medial-lateral rotation | 38.67 | 2.00 | 0.94 | 26.67 | -17.57 |
| m. pectoralis (cranial origin) | Glenohumeral | Abduction-adduction | 4.32 | -0.40 | 0.34 | 1.31 | -1.13 |
|  |  | Long axis rotation | 4.56 | 1.19 | 0.33 | 1.07 | -0.76 |
|  |  | Flexion-extension | 7.34 | -1.62 | 0.67 | 4.86 | -5.11 |
|  | Scapulocoracoid-clavicle-interclavicle | Medial-lateral rotation | 1.86 | -0.56 | 0.43 | 1.73 | -1.09 |
| m. pectoralis (caudal origin) | Glenohumeral | Abduction-adduction | 1.50 | -0.36 | 0.12 | 0.37 | -0.33 |
|  |  | Long axis rotation | 3.06 | 0.28 | 0.31 | 0.86 | -0.49 |
|  |  | Flexion-extension | 1.50 | -0.59 | 0.15 | 0.33 | -0.19 |
|  | Scapulocoracoid-clavicle-interclavicle | Medial-lateral rotation | 3.90 | 0.86 | 0.45 | 2.22 | -1.86 |
| m. triceps brachii longus | Glenohumeral | Abduction-adduction | 3.19 | 0.18 | 0.24 | 0.68 | -0.45 |
|  |  | Long axis rotation | 3.71 | 0.50 | 0.16 | 0.31 | -0.16 |
|  |  | Flexion-extension | 3.55 | -7.41 | 0.09 | 0.15 | -0.08 |
|  | Humeroradioulnar | Abduction-adduction | 2.35 | -0.27 | 0.17 | 0.33 | -0.24 |
|  |  | Long axis rotation | 1.72 | -0.39 | 0.13 | 0.37 | -0.23 |
|  |  | Flexion-extension | 1.15 | 0.16 | 0.08 | 0.16 | -0.14 |

**Supplementary Figure S1:** Muscle moment arms (MMAs) predicted for muscles crossing the scapulocoracoid-clavicle-interclavicle joint, from the initial model published as Fig. 4 in Regnault and Pierce (2018) (left graph), and from the revised muscle attachments using diceCT data from Regnault et al. (2020) (right graph). Note the revised attachment of m. biceps longus means it no longer crosses this joint in the model, and the moment arm thus changes from a peak value of -4.2mm to 0.


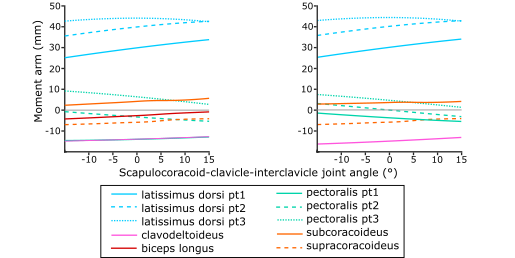


**Supplementary Figure S2:** Muscle moment arms (MMAs) predicted for muscles crossing the glenohumeral joint from the initial model published as Fig. 5 in Regnault and Pierce (2018) (left graph), and from the revised muscle attachments using diceCT data from Regnault et al. (2020) (right graph). Note that due to the revised model’s muscle pathways: a) m. triceps is unchanged but m. biceps brevis has become a flexor (positive), and both parts of mm. biceps have become internal rotators (positive), however these are all small compared to the larger MMAs (inferred principal action) in adduction; b) m. coracobrachialis has very mild changes; c) m. subcoracoideus has very mild changes; d) mm. teres minor and major have very mild changes; e) m. subscapularis is essentially unchanged; f) mm. supraspinatus and infraspinatus have some mild changes; g) m. latissimus dorsi has some mild changes; h) m. pectoralis has changes that result in the cranial portion (part 1) acting more similarly to the rest of the muscle, as an internal humeral rotator and adductor; i) some changes at the deltoid muscles, particularly m. clavodeltoideus, but similar overall.

(next page)


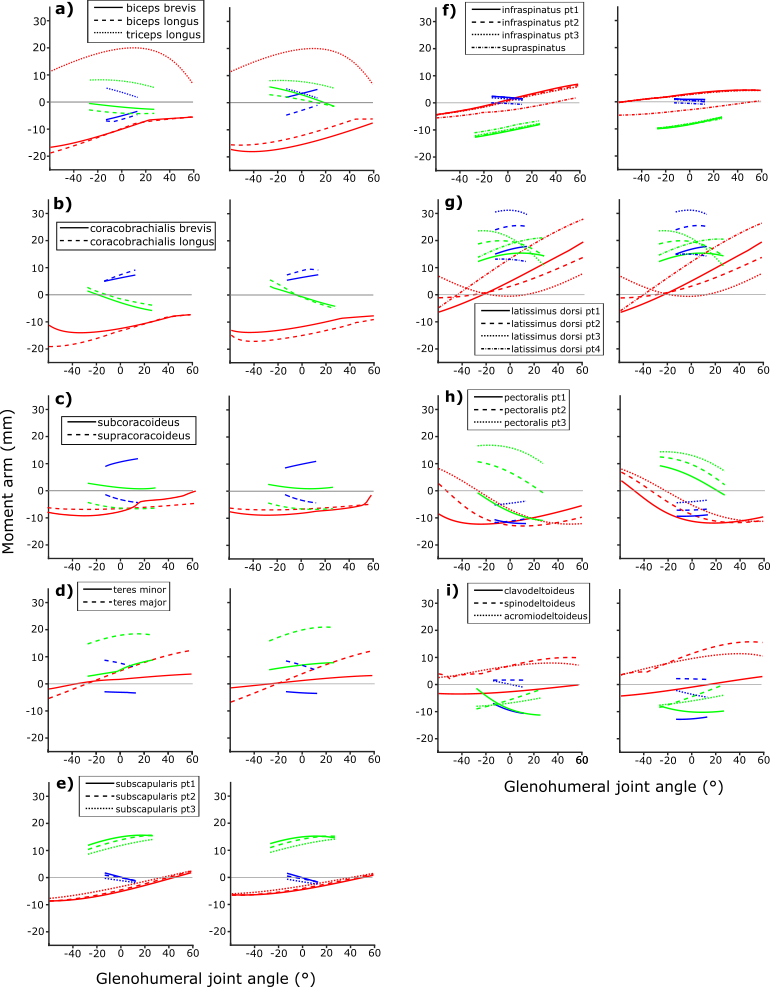


**Supplementary Figure S3:** Muscle moment arms predicted for muscles crossing the humeroradioulnar joint from the initial model published as Fig. 6 in Regnault and Pierce (2018) (left graph), and from the revised muscle attachments using diceCT data from Regnault et al. (2020) (right graph). Note that due to the revised model’s muscle pathways: a) m. biceps brevis flexion MMAs are smaller, but the other MMAs for mm. biceps and triceps brachii are relatively similar; b) m. triceps brachii MMAs are unchanged.


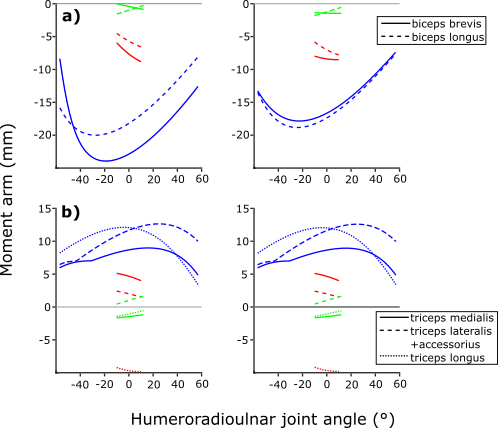


**Supplementary Figure S4:** Three-dimensional (3D) joint ROM at the glenohumeral joint (top row) and humeroradioulnar joint (bottom row), broken down into individual specimen trial set contributions to the total pooled ROM shown in Figure 4: specimen number 44 Left side in blue, number 46 Left side in orange, 46 Right side in green, 48 Left side in red, and 48 Right side in purple. Note that axis scale differs between plots.


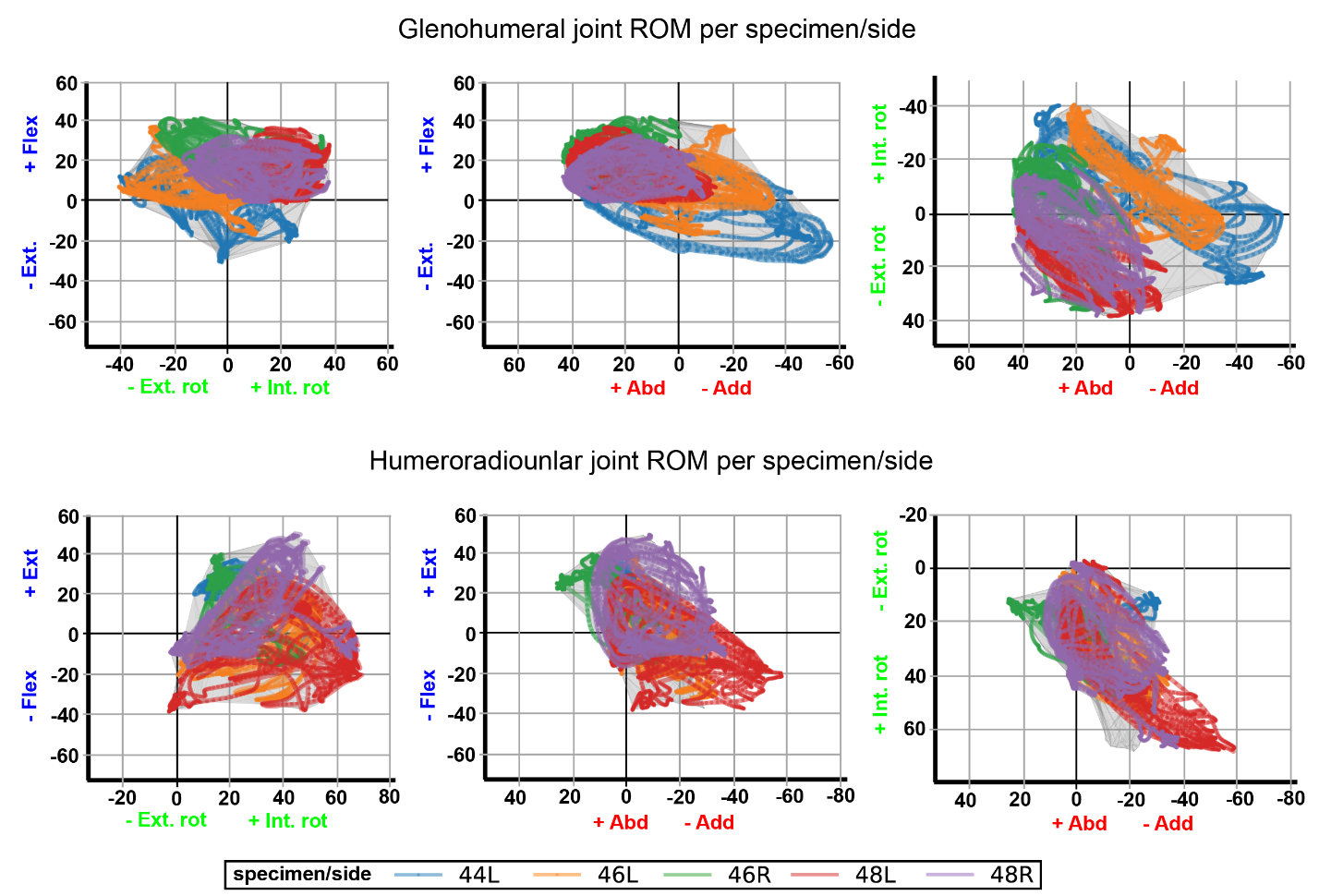


**Supplementary Figure S5:** Three-dimensional (3D) MMAs for m. biceps brachii and m. triceps brachii at the humeroradioulnar joint, plotted in cosine-corrected ROM space for the SIMM model (using partial velocity) and Maya experimental data (using the geometric method). MMA sign is indicated by point colour (positive values are purple, negative values are orange) with colour intensity scaled to relative MMA magnitude as described in the Methods. ABAD = abduction-adduction; LAR = long-axis rotation (internal-external rotation); FE = flexion-extension.

(next page)


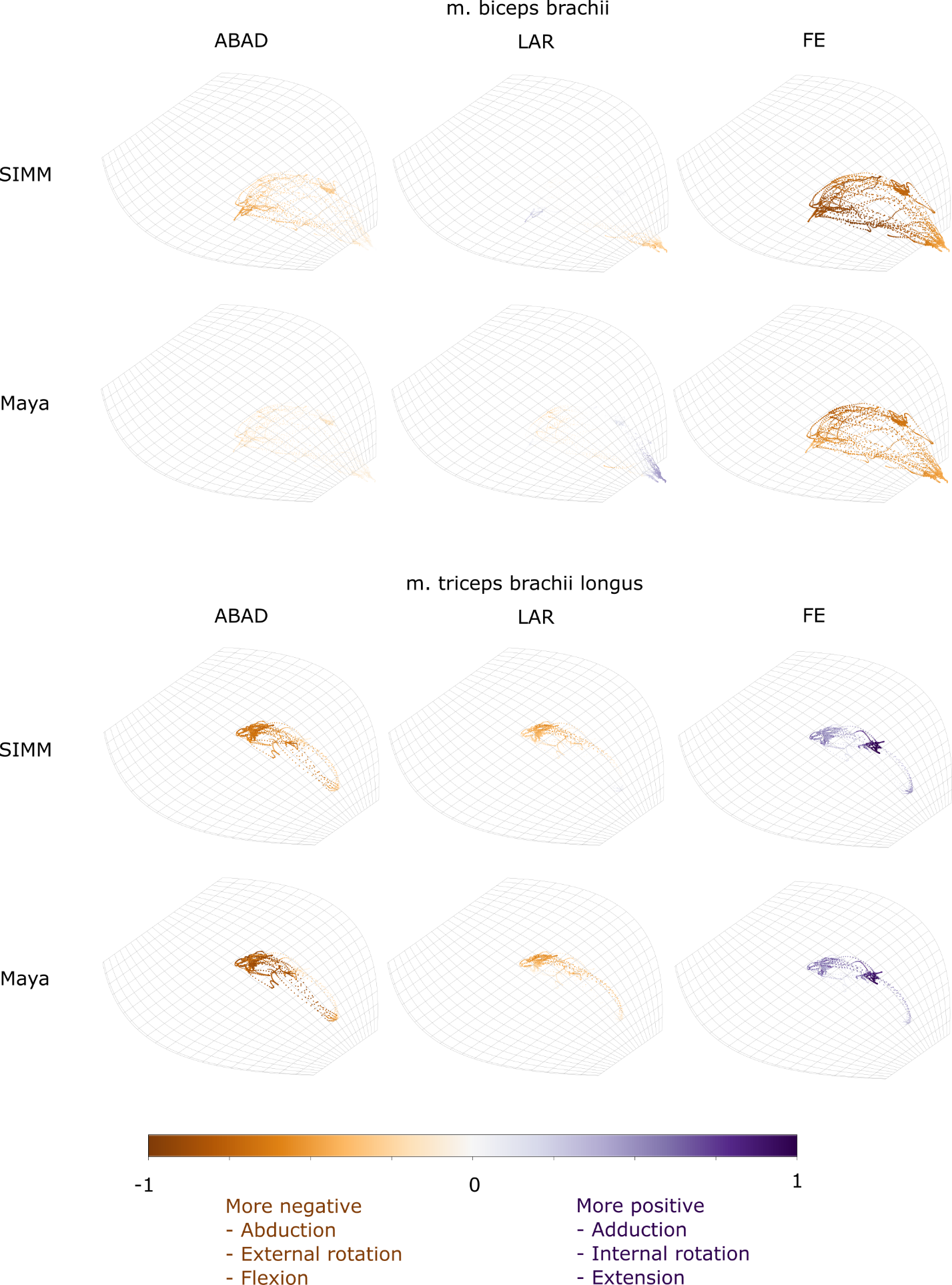


**Supplementary Figure S6:** Updated SIMM model muscle torques for individual muscles crossing the scapulocoracoid-clavicle-interclavicle joint.


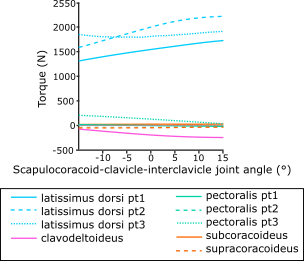


**Supplementary Figure S7:** Updated SIMM model muscle torques for individual muscles crossing the glenohumeral joint. Abbreviations: BI-B and BI-L = m. biceps brachii brevis and longus; CB-B and CB-L = m. coracobrachialis brevis and longus; SUBCOR = m. subcoracoideus; SUPCOR = m. supracoracoideus; TMN = m. teres minor; TMJ = m. teres major; SUBSCP = m. subscapularis (modelled as three parts); INFSP = m. infraspinatus (modelled as three parts); SUSP = m. supraspinatus (modelled as three parts); LAT = m. latissimus dorsi (modelled as four parts); PECT = m. pectoralis (modelled as three parts); CLAV-D = m. clavodeltoideus; SPN-D = m. spinodeltoideus; ACR-D = m. acromiodeltoideus.


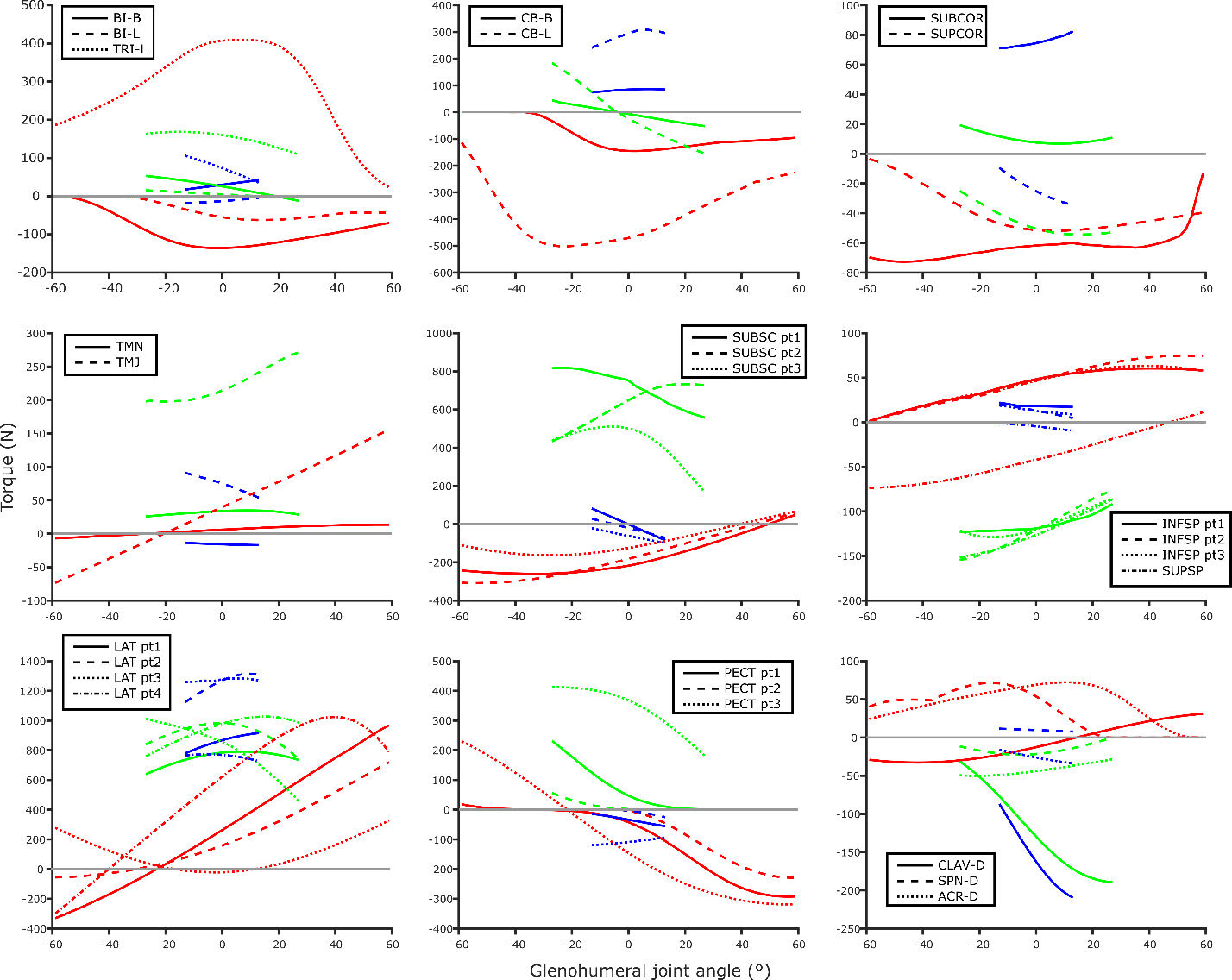


**Supplementary Figure S8:** Updated SIMM model muscle torques for individual muscles crossing the humeroradioulnar joint. a) biceps and b) triceps.


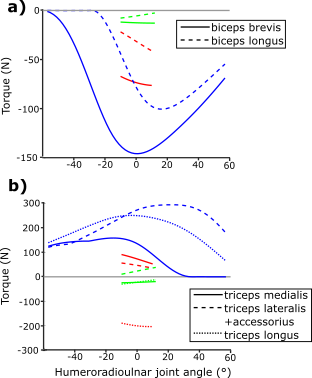


**Supplementary Figure S9**: Visualisation for how geometric moment arms were calculated. a) Per-axis 3D vector moment arms (e.g., $\vec{r_{x}}$) are calculated by finding the shortest distance between two skew lines: a unit vector (e.g., $\hat{x}$) representing a joint axis, and a muscle vector $\vec{F}$ running between implanted marker beads. b) The shortest distance between any two skew lines is always perpendicular to both lines. c) The vector moment arm $\vec{r}$ is equally valid for any force vector (e.g., $\vec{F}'$,$\vec{F}''$) that intersects with it at the same point p. To obtain an accurate scalar moment arm $r_{\theta}$, the direction of the force vector must be specified by scaling ||$\vec{r}$|| by the fraction of ||$\vec{F}$||that lies in plane x (red circle).


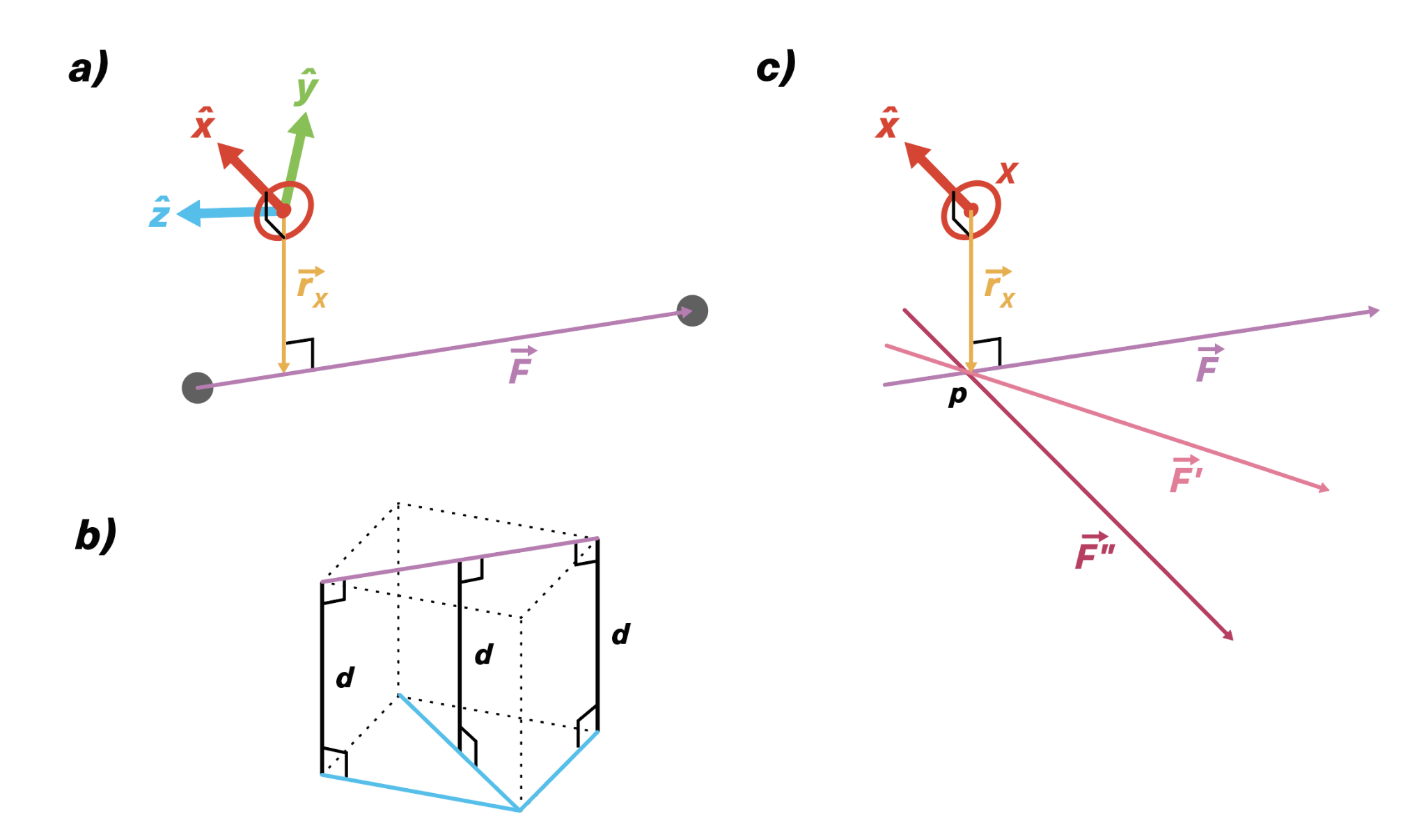

Supplement: Supplementary file 2 [file DataSheet1.docx]
